# Single-cell polygenic risk scores dissect cellular and molecular heterogeneity of complex human diseases

**DOI:** 10.1038/s41587-025-02725-6

**Published:** 2025-07-25

**Authors:** Sai Zhang, Hantao Shu, Jingtian Zhou, Jasper Rubin-Sigler, Xiaoyu Yang, Yuxi Liu, Johnathan Cooper-Knock, Emma Monte, Chenchen Zhu, Sharon Tu, Han Li, Mingming Tong, Joseph R. Ecker, Justin K. Ichida, Yin Shen, Jianyang Zeng, Philip S. Tsao, Michael P. Snyder

**Affiliations:** 1 Department of Epidemiology, University of Florida, Gainesville, FL USA; 2 Departments of Biostatistics & Biomedical Engineering, UF Genetics Institute, University of Florida, Gainesville, FL USA; 3 Department of Genetics, Center for Genomics and Personalized Medicine, Stanford University School of Medicine, Stanford, CA USA; 4 VA Palo Alto Healthcare System, Palo Alto, CA USA; 5 Institute for Interdisciplinary Information Sciences, Tsinghua University, Beijing, China; 6 Arc Institute, Palo Alto, CA USA; 7 Genomic Analysis Laboratory, The Salk Institute for Biological Studies, La Jolla, CA USA; 8 Bioinformatics and Systems Biology Program, University of California, San Diego, La Jolla, CA USA; 9 Department of Stem Cell Biology and Regenerative Medicine, Eli and Edythe Broad Center for Regenerative Medicine and Stem Cell Research, University of Southern California, Los Angeles, CA USA; 10 Institute for Human Genetics, University of California, San Francisco, San Francisco, CA USA; 11 Sheffield Institute for Translational Neuroscience, University of Sheffield, Sheffield, UK; 12 Howard Hughes Medical Institute, The Salk Institute for Biological Studies, La Jolla, CA USA; 13 Department of Neurology, Weill Institute for Neurosciences, University of California, San Francisco, San Francisco, CA USA; 14 School of Engineering, Research Center for Industries of the Future, Westlake University, Hangzhou, China; 15 Department of Medicine, Stanford University School of Medicine, Stanford, CA USA

**Keywords:** Machine learning, Genomics, Diseases, Gene regulation, Genetic association study

## Abstract

Polygenic risk scores (PRSs) predict an individual’s genetic risk for complex diseases, yet their utility in elucidating disease biology remains limited. We introduce scPRS, a graph neural network-based framework that computes single-cell-resolved PRSs by integrating reference single-cell chromatin accessibility profiles. scPRS outperforms traditional PRS approaches in genetic risk prediction, as demonstrated across multiple diseases including type 2 diabetes, hypertrophic cardiomyopathy, Alzheimer disease and severe COVID-19. Beyond risk prediction, scPRS prioritizes disease-critical cells and, when combined with a layered multiomic analysis, links risk variants to gene regulation in a cell-type-specific manner. Applied to these diseases, scPRS fine-maps causal cell types and cell-type-specific variants and genes, demonstrating its ability to bridge genetic risk with cell-specific biology. scPRS provides a unified framework for genetic risk prediction and mechanistic dissection of complex diseases, laying a methodological foundation for single-cell genetics.

## Abstract (web summary)

A graph neural network framework enables individualized genetic risk prediction at the single-cell level.

## Main

Polygenic risk score^1^ (PRS), also known as polygenic score^2^, is a widely used approach to predict quantitative traits and disease risk on the basis of an individual’s genetic makeup. The method is built upon genetic variants, including single-nucleotide polymorphisms (SNPs) and small insertions and deletions (indels) that are common (minor allele frequency (MAF) > 5%) in the population. PRS is a critical component of precision genomic medicine and has promise in versatile utilities^3^, such as health management, disease screening and therapeutic intervention. Traditionally, PRS computation involves a linear model that sums the genotypes of selected variants, with each variant weighted according to its effect size as estimated by a genome-wide association study^4^ (GWAS). The clumping and thresholding (C+T) method serves as the basis of constructing PRSs; however, other advanced approaches^5–8^ have also been developed to enhance prediction by considering nuanced genetic architectures. Complex diseases exhibit notable cellular heterogeneity, involving multiple tissues or cell types in their pathogenesis^9^. Risk variants, particularly noncoding ones, can influence disease susceptibility and phenotypic variability through diverse cellular and molecular processes^10–12^. However, these multiple layers of complexity have been oversimplified in conventional modeling, substantially limiting the predictive power and interpretability of PRS^13^.

In recent years, single-cell sequencing has emerged as a potent tool to dissect cellular and molecular heterogeneity across different tissues and conditions^14^, offering unprecedented opportunities to explore genome function at high resolution. Single-cell profiling data from healthy tissues provide high-resolution annotations of the baseline genome function in which genetic variants are involved. Incorporating functional annotations into PRS calculation will remove confounders such as linked nonfunctional variants, better characterize a disease’s genetic architecture and, therefore, improve the predictive accuracy and generalization. This has been demonstrated elsewhere^15^, including our latest study^16^. Moreover, the interpretability of PRS can be considerably enhanced by incorporating functional information, adding biological discovery functionality to predictive methods.

To bridge this gap, we propose a strategy that unifies genetics and single-cell genomics, named single-cell genetics^17^, to study disease genetics at single-cell resolution. In particular, we introduce scPRS, a graph neural network^18^ (GNN)-based framework that enables individualized genetic risk prediction at the single-cell level. scPRS leverages the GNN to construct genetic risk score by drawing insights from reference single-cell chromatin accessibility measured by single-cell or single-nucleus sequencing assay for transposase-accessible chromatin^19^ (scATAC-seq or snATAC-seq). scATAC-seq or snATAC-seq maps single-cell-resolved candidate *cis*-regulatory elements^20^ (cCREs), which are specific DNA regions that potentially regulate the transcription of nearby genes. Beyond enhanced disease prediction, scPRS is empowered with fine-grained model interpretability, which allows for systematic discovery of cell types and cell-type-specific gene-regulatory programs underpinning diseases.

We performed extensive simulation experiments to demonstrate the effectiveness and robustness of scPRS in identifying phenotype-relevant cells. We applied scPRS to four diseases—type 2 diabetes (T2D), hypertrophic cardiomyopathy (HCM), Alzheimer disease (AD) and severe COVID-19—and showcased its superior predictive performance compared to traditional PRS methods. Through model interpretation, scPRS identified known disease-critical cell types as well as previously uncharacterized cell populations. scPRS-powered functional analysis further fine-mapped candidate causal variants, cCREs and target genes within specific cell types, revealing a cell-type-specific landscape of genetic regulation. Using drug perturbation data, we validated our scPRS-nominated HCM genes, showing that the suppression of these genes in diseased cardiomyocytes (CDMs) was rescued by mavacamten (a US Food and Drug Administration (FDA)-approved HCM drug) treatment. Supported by experiments, we identified a new role of the AD risk variant rs7922621 in downregulating *ANXA11* and *TSPAN14*, specifically in microglia. We also demonstrated the negative effect of suppressing these genes on microglial phagocytosis. Taken together, scPRS offers a unified approach that encompasses GNN modeling and GNN-inspired downstream analysis for simultaneous disease prediction and biological discovery, establishing the methodological foundation for single-cell genetics.

## Results

### Overview of scPRS

**Fig. 1:**
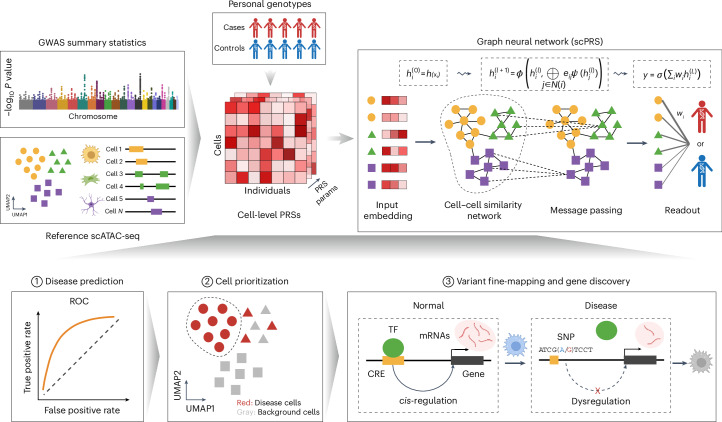
Overview of scPRS and its applications. For a given disease, scPRS first leverages GWAS summary statistics obtained from the discovery cohort and the reference scATAC-seq or snATAC-seq dataset to calculate single-cell-level PRSs with different parameters for individuals in the target cohort. Next, scPRS embeds and propagates cell-level PRSs over the cell–cell similarity network using a GNN. The final readout combines smoothed PRSs from all cells to predict the disease risk. scPRS is trained to minimize the loss between predicted and true disease labels. The trained model can be used to (1) predict disease risk for unseen individuals; (2) prioritize disease-relevant cells and cell types; and (3) fine-map disease risk variants, genes and disrupted genetic regulation in specific cell types. UMAP, uniform manifold approximation and projection. The schematic was created using BioRender.com.

The design principle of scPRS is to leverage single-cell epigenome profiling to rationalize the calculation of PRS. The approach begins with deconvoluting traditional PRS within individual cells on the basis of their chromatin accessibility profiled by scATAC-seq, followed by the integration of decomposed single-cell-level PRSs into a final score capitalizing on cell–cell similarities (Fig. 1 and Methods). In particular, using GWAS summary statistics derived from a disease cohort (referred to as the discovery cohort) and an scATAC-seq dataset of healthy tissue pertinent to the disease (referred to as the reference scATAC-seq dataset), we compute a conditioned PRS for each individual within our target cohort (independent with the discovery cohort) and for each reference cell, in which we mask out genetic variants located outside open chromatin regions captured in that specific cell. Recognizing the sparsity of scATAC-seq data, scPRS further refines per-cell PRS features using a GNN^21^. This GNN operation serves the dual purpose of denoising raw PRS features while capturing nonlinear relationships between genetic signals and the cellular epigenome. In the final step, scPRS aggregates smoothed single-cell-level PRSs and yields a final disease risk score. The interpretability of scPRS is achieved by the learned model weights accompanied with single cells that indicate the contribution of different cells to the disease risk.

The functionalities of scPRS are exemplified by three downstream tasks. First, scPRS predicts disease risk for unseen individuals solely on the basis of their genotypes (Fig. 1, step 1). Second, scPRS prioritizes single cells that are relevant to the disease, overcoming the resolution constraint of predefined cell clusters (Fig. 1, step 2). Third, integrated with a multiomic approach, scPRS fine-maps causal variants, genes and genetic regulation within prioritized cell types (Fig. 1, step 3).

### Evaluation of scPRS using simulations

**Extended Data Fig. 1:**
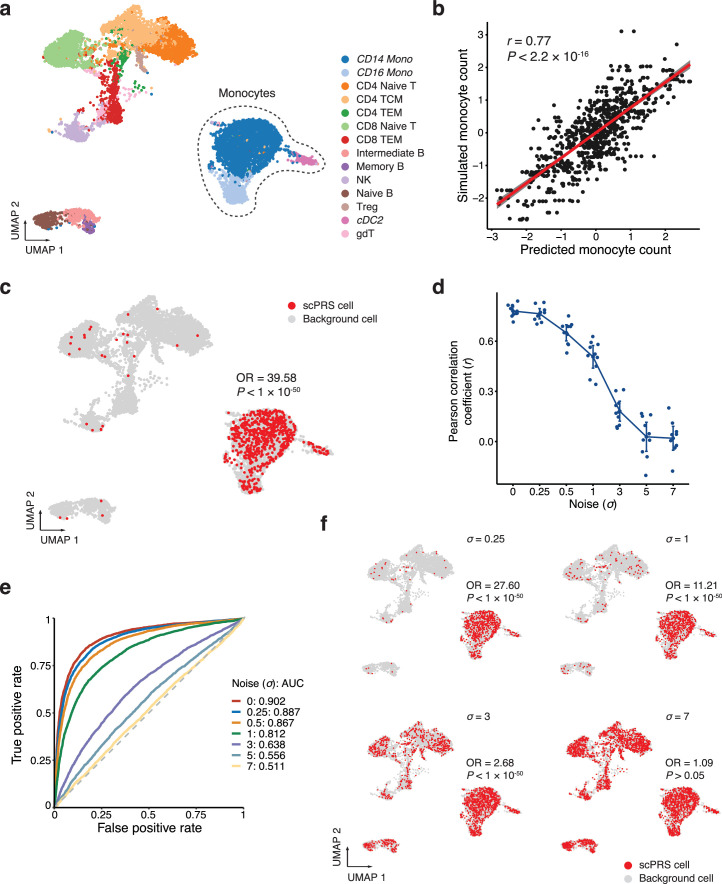
Assessing performance of scPRS using simulations. **a**, The uniform manifold approximation and projection (UMAP) plot of the human peripheral blood mononuclear cell (PBMC) scATAC-seq dataset. Cell clusters with less than 150 cells are not shown. Monocyte subtypes are highlighted in italic. Mono, monocyte; TCM, memory T cell; TEM, effector memory T cell; NK, natural killer cell; Treg, regulatory T cell; cDC2, conventional type 2 dendritic cell; gdT, gamma-delta T cell. **b**, Pearson correlation between simulated and predicted monocyte counts (*n* = 10 repeats). *P*-value by two-sided Pearson correlation. The linear regression line and 95% confidence interval (CI) are annotated in the red line and gray shaded area, respectively. **c**, Monocyte-count-relevant cells prioritized by scPRS (in red). Odds ratio and *P*-value by two-sided Fisher’s exact test. OR, odds ratio. **d**, Pearson correlation between simulated and predicted monocyte counts (*n* = 10 repeats) in different noise settings. The mean and 95% CI are annotated in the dot and error bar, respectively. *σ*, standard deviation. **e**, The receiver operating characteristic (ROC) curves for cell prioritization in different noise settings, wherein monocytes were labeled as “1” and other cells were labeled as “0”. AUC, the area under the curve. **f**, Monocyte-count-relevant cells prioritized by scPRS (in red) in different noise settings. Odds ratio and *P*-value by two-sided Fisher’s exact test.

We first performed simulation experiments to evaluate the capacity of scPRS in identifying phenotype-relevant cells. Assuming that the trait ‘monocyte count’ is fully determined by genetic variants located within monocyte-specific open chromatin regions^22^, we simulated monocyte counts for individuals of a genotyped cohort^23^ (*n* = 401). We then asked whether we could use scPRS to recapitulate monocytes as the causal cell type. Specifically, we used a reference scATAC-seq dataset^24^ (Extended Data Fig. 1a) of human peripheral blood mononuclear cells (PBMCs) to identify monocyte-specific peaks (Methods). On the basis of a monocyte count GWAS^22^ defining variant effect sizes, we simulated the monocyte count for each individual by calculating the C+T PRS using only variants located within monocyte-specific peaks (Methods). Next, we trained an scPRS model to predict simulated monocyte counts from cell-level PRSs computed on all PBMCs. We observed that scPRS predictions were significantly correlated with simulated monocyte counts (*r* = 0.77, *P* < 2.2 × 10^−16^, Pearson correlation; Extended Data Fig. 1b). The cells prioritized by scPRS (Methods) were significantly enriched within monocytes (*Z* = 39.58, *P* < 1 × 10^−50^, two-sided Fisher’s exact test; Extended Data Fig. 1c), demonstrating that scPRS captured causal cells.

Human phenotypes such as complex diseases can be influenced by various nongenetic factors, including environmental and lifestyle factors^25^. Additionally, the measurement of phenotypes often carries inherent noise. Therefore, it is important to assess the robustness of scPRS by introducing noise and randomness into the simulation (Methods). As expected, we observed a progressive reduction in predictive performance as we introduced larger amounts of noise (Extended Data Fig. 1d). Notably, scPRS sustained its ability in uncovering monocytes even in the presence of considerable noise terms (Extended Data Fig. 1e,f and Supplementary Fig. 1a). For example, when we introduced a noise term with the same amount of variance (*σ* = 1) as that of the simulated phenotype, scPRS still accurately identified monocytes (area under the curve (AUC) = 0.812; Extended Data Fig. 1e); the enrichment of monocytes persisted even when three times the amount of variance was added (*σ* = 3; *Z* = 2.68, *P* < 1 × 10^−50^, two-sided Fisher’s exact test; Extended Data Fig. 1f).

We further introduced peak noise into simulation by replacing a proportion of randomly selected monocyte-specific peaks with non-monocyte-specific peaks. Using these mixed peaks, we generated noisy monocyte counts for individuals. We then assessed whether scPRS could still identify monocytes from the noisy data. We found that scPRS was able to identify monocytes with peak noise levels up to 90% (Supplementary Fig. 1b,c). We also tested different model hyperparameter settings and observed no significant variation in predictive performance (Supplementary Fig. 1d). All these results demonstrate the robustness of scPRS against different sources of noise, randomness and model settings.

Lastly, we conducted a negative control experiment by excluding monocyte-related cells, including monocytes and cells containing more than 40 monocyte-specific peaks (~1% of all peaks used for simulating monocyte counts) from the PBMC dataset. Unsurprisingly, the predictive performance of scPRS was significantly reduced (*r* = 0.488 (mean) ± 0.085 (s.d.); Supplementary Fig. 1e) compared to scPRS trained on the full dataset. Moreover, scPRS exhibited increased nonspecificity in prioritizing monocyte-count-relevant cells (Supplementary Fig. 1f), showing a similar saturation pattern in large-noise scenarios (Extended Data Fig. 1f).

### scPRS accurately predicts diseases

We applied scPRS to multiple diseases, including T2D, HCM, AD and severe COVID-19. We used UK Biobank^26^ (UKBB) data to construct target cohorts for T2D and AD and our in-house whole-genome sequencing (WGS) data^27^ for HCM (Methods). The severe COVID-19 target cohort was constructed on the basis of the Veterans Affairs (VA) Million Veteran Program^28^ (MVP) WGS dataset (Methods). The discovery GWAS dataset^29–32^ was carefully chosen to ensure nonoverlap with the target cohort for each disease. Multiple reference scATAC-seq datasets of disease-relevant tissues were used, including the pancreas^33^ for T2D, left ventricle^34^ for HCM, frontal cortex^35^ for AD and lung^34^ for severe COVID-19 (Methods).

For benchmarking, we used six well-established PRS methods: C+T (implemented by PLINK^36^), LDpred2 (including LDpred2-inf, LDpred2-grid, and LDpred2-auto)^5^, Lassosum^7^ and PolyPred^37^ (Methods). Among these baseline methods, PolyPred uses functional annotations to compute prior causal probabilities of variants^38^, for which we used scATAC-seq peaks as the annotation to ensure a fair comparison. To examine the predictability of nonpeak and nongenetic factors, we also built a C+T PRS model on the basis of variants situated beyond open chromatin regions and a logistic regression (LR) model using individual’s age, sex and the first ten principal components (PCs) as input features (Methods).

**Fig. 2:**
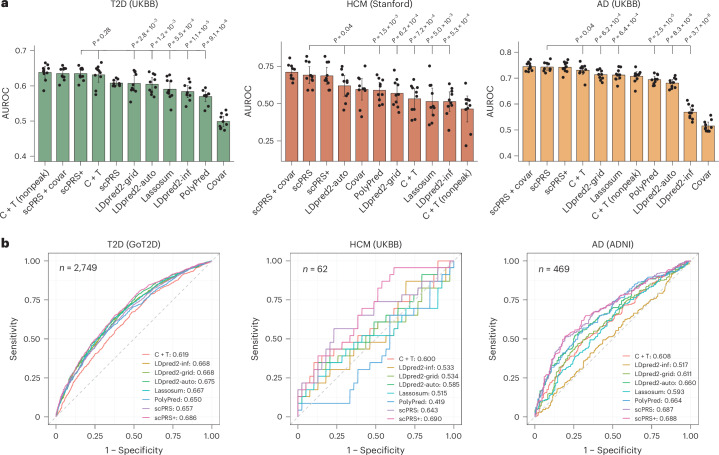
Predictive performance comparison between scPRS and baseline methods. **a**, Bar plots of AUROC values of different models. The training and testing procedure was conducted for ten repeats with different random seeds. Training, validation and test dataset splits were kept identical across different methods to ensure a fair comparison. scPRS+, scPRS model integrating nonpeak PRSs; scPRS+covar, scPRS model integrating nonpeak PRSs and covariates (that is, age, sex and first ten PCs); C+T (nonpeak), LR model of nonpeak C+T PRSs; Covar, LR model of covariates. Performance comparison was conducted using a one-sided paired *t*-test. The mean and 95% confidence interval (CI) are annotated using the bar plot and error bar, respectively. **b**, ROC curves of different models evaluated on independent target cohorts. The performance of a random predictor is shown by the dashed gray line.

Remarkably, scPRS-based methods consistently outperformed all baseline PRS approaches in all diseases (Fig. 2a and Supplementary Fig. 2a,b). In particular, for HCM, AD and severe COVID-19, scPRS achieved superior predictive performance evaluated by both the area under the receiver operating characteristic curve (AUROC; HCM, 0.692 ± 0.079; AD, 0.743 ± 0.017; severe COVID-19, 0.591 ± 0.029) and the area under the precision–recall curve (AUPRC; HCM, 0.781 ± 0.062; AD, 0.751 ± 0.035; severe COVID-19, 0.281 ± 0.034) compared to all baseline PRS methods (adjusted *P* < 0.1, Benjamini–Hochberg (BH) correction; Fig. 2a and Supplementary Fig. 2a,b), except for C+T and LDpred2-auto, which yielded comparable AUPRC values in some cases.

For T2D, scPRS presented performance comparable to other methods (AUROC, 0.608 ± 0.009; AUPRC, 0.598 ± 0.032; Fig. 2a and Supplementary Fig. 2b). Integrating nonpeak C+T PRSs into the scPRS model (referred to as scPRS+; Methods) further boosted its performance (AUROC, 0.635 ± 0.018; AUPRC, 0.633 ± 0.036), outperforming all baseline methods (adjusted *P* < 0.1, BH correction; Fig. 2a and Supplementary Fig. 2b), except for C+T where the AUROC remained comparable. These results suggest that the variants located outside pancreas cCREs, such as protein-coding^39^ and splicing^40^ variants, or variants within cCREs specific to other tissues^41^ may also contribute to T2D susceptibility. This is also supported by the observation that a predictor built solely on nonpeak PRSs (referred to as nonpeak C+T) performed best among all methods (AUROC, 0.638 ± 0.023; AUPRC, 0.633 ± 0.039; Fig. 2a and Supplementary Fig. 2b).

We also constructed peak PRSs across different cell types annotated in the scATAC-seq datasets (Methods). scPRS outperformed all single-cell-type and multi-cell-type PRSs for all diseases (Supplementary Fig. 3), underscoring the advantage of single-cell-resolved modeling in disease prediction.

The covariate models exhibited limited predictive power for T2D and AD (Fig. 2a and Supplementary Fig. 2b) because of the fact that we matched age, sex and population between cases and controls in constructing the target cohorts. Not surprisingly, the predictive performance reached a peak for all diseases after integrating all other factors, including nonpeak PRSs and covariates, into the scPRS model (referred to as scPRS+covar; Fig. 2a and Supplementary Fig. 2a,b).

We tested the use of alternative scATAC-seq datasets in scPRS, including those from a different study^42^ (for AD), a different donor (for HCM) and a different sampling (for T2D). We found that scPRS yielded comparable predictive performance (Supplementary Fig. 4a,b), demonstrating its robustness against distinct choices of reference single-cell datasets. To examine the impact of cell numbers, we compared the predictive performance of T2D scPRS models using different numbers of cells randomly sampled from the pancreas scATAC-seq dataset. We observed that scPRS exhibited moderately stable predictive performance across a broad range of cell numbers (Supplementary Fig. 4c), with an increase in performance as more cells were sampled. We also assessed the impact of input PRS choices. In particular, we randomly removed input PRSs, in which a certain proportion of randomly selected PRS features were set to zero for all samples in each training–testing procedure. We then evaluated the predictive performance of scPRS across different dropout rates. scPRS yielded stable predictive performance with only a slight decrease as dropout rates increased up to 70% (Supplementary Fig. 4d), whereas performance was substantially reduced at higher dropout rates.

As a negative control, we chose PBMCs as an unrelated system for T2D. scPRS trained on PBMC scATAC-seq data presented inferior predictive performance compared to the model trained on the pancreas data (Supplementary Fig. 5a), highlighting the importance of choosing reference single-cell data from disease-relevant systems or tissues in scPRS.

Lastly, we sought to evaluate scPRS on independent target cohorts. For T2D, we used the Genetics of T2D Consortium^39^ (GoT2D) genotype dataset as the independent cohort; for HCM, because the discovery GWAS was performed on UKBB European (EUR) samples, we constructed an independent cohort comprising non-EUR HCM samples and matched controls from UKBB; for AD, we used the AD Neuroimaging Initiative^43^ (ADNI) WGS dataset. We trained scPRS models on the basis of the original target cohorts and all PRS methods were tested on the new independent target cohorts. Notably, scPRS still outperformed all baseline methods for HCM and AD (Fig. 2b and Supplementary Fig. 2c). Similarly, scPRS+ further improved the prediction for T2D, surpassing all other baseline PRS approaches (Fig. 2b and Supplementary Fig. 2c). Interestingly, for HCM, even when scPRS was trained on EUR samples, it performed comparably for non-EUR samples (AUROC, 0.692 (EUR) versus 0.643 (non-EUR); Fig. 2b and Supplementary Fig. 2c), suggesting its portability across different populations, although further validation with additional data is needed.

### scPRS prioritizes disease-relevant cells

Next, we sought to examine the disease–cell association using scPRS. For each disease, we first trained 100 scPRS models with different random seeds based on the entire target cohort and then prioritized cells whose model weights consistently exceeded those of background cells, designating them as disease-relevant cells (Methods). We also harnessed the knowledge of annotated cell types to facilitate biological interpretation (Methods).

#### T2D

**Fig. 3:**
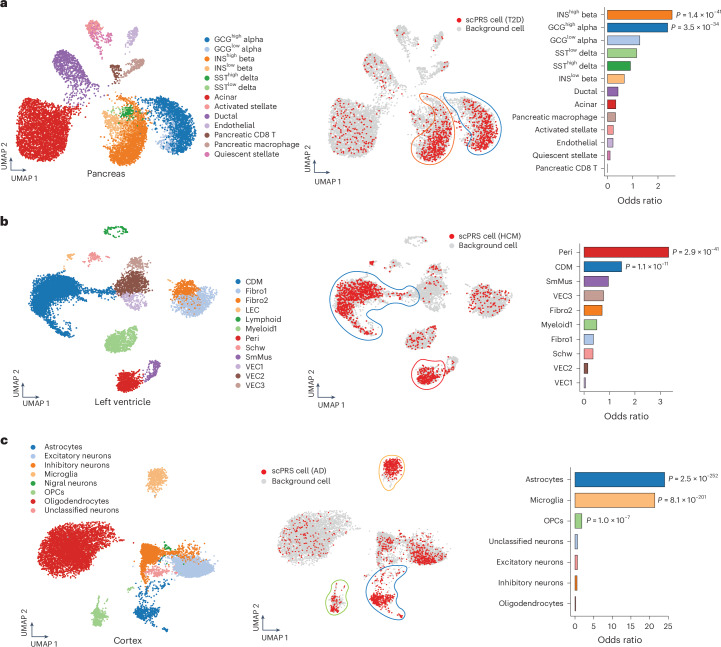
Disease-critical cells identified by scPRS. **a**–**c**, Disease-critical cells identified by scPRS for T2D (**a**), HCM (**b**) and AD (**c**). Left, scATAC-seq or snATAC-seq datasets used by scPRS, along with annotated cell types. Middle, disease-relevant cells prioritized by scPRS (in red). Cell clusters enriched with scPRS-prioritized cells are highlighted in closed curves with corresponding cell type colors. Right, enrichment of scPRS-selected disease cells within each cell type. ORs and *P* values were determined using a one-sided Fisher’s exact test. Cell type abbreviations: Fibro, fibroblast; LEC, lymphatic endothelial cell; Peri, pericyte; Schw, Schwann cell; SmMus, smooth muscle cell; VEC, vascular endothelial cell. For robustness, small cell clusters with fewer than 150 cells were excluded from analysis and visualization for all diseases.

There were 14 cell types identified in the human pancreas^33^ (Fig. 3a, left, and Methods), among which two hormone-high cell types (namely, GCG^high^ alpha cells and INS^high^ beta cells) were significantly enriched with scPRS-selected cells (adjusted *P* < 0.1, BH correction; Fig. 3a). The original study^44^ that generated the pancreas snATAC-seq dataset had linked INS^high^ and INS^low^ beta cells to T2D risk using the stratified linkage disequilibrium (LD) score regression^45^ (sLDSC). As another benchmark, we applied SCAVENGE^46^, a computational method that also enables single-cell-resolved cell prioritization, to the same data (Methods). In addition to GCG^high^ alpha cells and INS^high^ beta cells, SCAVENGE prioritized GCG^low^ alpha cells (adjusted *P* < 0.1, BH correction; Supplementary Fig. 6a). In comparison, cells selected by PBMC-based T2D scPRS exhibited nonspecificity across cell types (Supplementary Fig. 5b).

While pancreatic beta cell dysfunction and cell death are known as key processes in the development of T2D (ref. ^47^), it is increasingly evident that T2D may result from defects in multiple cell types^48^. Notably, the alpha cell, which serves as the counterpart to the beta cell and is responsible for producing glucagon, has been increasingly recognized for its role in T2D pathogenesis^49–51^. Single-cell profiling further revealed the diversity within islet endocrine cells, spanning from fine-grained cell states to a continuous spectrum^44^. Our findings, coupled with prior research^44,52^, underscore the complexity of T2D pathogenesis involving multiple cell types within the pancreatic islets.

#### HCM

In the human left ventricle, a total of 17 cell types were identified (Fig. 3b, left, and Methods). Among these, two cell types, including CDMs and pericytes, presented significant enrichment with scPRS-selected cells (adjusted *P* < 0.1, BH correction; Fig. 3b). As comparison, we found no genetic enrichment within snATAC-seq peaks of all left-ventricle cell types using sLDSC (Supplementary Fig. 6b and Methods). SCAVENGE also linked CDMs to HCM (adjusted *P* < 0.1, BH correction) but enrichment within pericytes was not observed (Supplementary Fig. 6c). CDMs, the primary cell type involved in the process of hypertrophy and thickening of heart muscle, have a pivotal role in HCM pathogenesis^53^. Pathogenic mutations disrupt the normal function of CDMs, leading to structural and functional abnormalities^53^, such as myocardial hypertrophy and fibrosis, contractile dysfunction and arrhythmias. Our scPRS prediction not only reinforces the association between CDM dysfunction and HCM but also extends this connection from protein function to noncoding gene regulation.

Cardiac pericytes interact with endothelial cells through both physical and paracrine mechanisms and are integral in maintaining cardiac and vascular homeostasis^54^. Despite being relatively understudied, the loss and dysfunction of pericytes have been associated with cardiomyopathy^55–57^. Our results confirm this connection and shed light on the potential causal involvement of pericytes in cardiac hypertrophy. Importantly, this link would not have been identified with either sLDSC or SCAVENGE.

#### AD

Eight major cell types were identified in the human cortex^35^ (middle frontal and superior and middle temporal gyri; Fig. 3c, left, and Methods), among which three cell types were significantly enriched with scPRS-prioritized cells (adjusted *P* < 0.1, BH correction; Fig. 3c), including microglia, astrocytes and oligodendrocyte progenitor cells (OPCs). It is noteworthy that the original study^35^ that generated the brain scATAC-seq dataset linked only microglia to AD using sLDSC. Applying SCAVENGE to the same data revealed the same set of AD-relevant cell types as scPRS (adjusted *P* < 0.1, BH correction; Supplementary Fig. 6d).

The relationship between microglia and AD has been well established in the literature^58^. Microglia have diverse roles, including immune response, phagocytosis and synapsis modulation, contributing extensively to the development and progression of AD pathology. Moreover, genetic studies consistently prioritize microglia as the most prominent brain cell type associated with AD^59,60^. In recent years, accumulating evidence has underscored the essential role of astrocytes in AD pathogenesis through their reactivation or dysfunction^61,62^. Additionally, latest research has linked OPCs to AD, likely because of its function in immune modulation and remyelination^63^. Our results reinforce these findings and offer further insights into the cellular heterogeneity of AD pathogenesis.

#### Severe COVID-19

scPRS-prioritized cells were significantly enriched in macrophages, natural killer (NK) cells and monocytes (adjusted *P* < 0.1, BH correction; Supplementary Fig. 6e). Dysregulated activation of macrophages contributes to tissue damage and disease progression through excessive cytokine production^64–66^. NK cells, crucial for early defense against viral infections, may exacerbate the cytokine storm when impaired^67–70^. Monocytes, as precursors to macrophages, have also been linked to severe COVID-19 because of their role in inflammation and tissue damage^71–73^. In particular, monocytes were also prioritized by SCAVENGE^46^ for severe illness.

Of note, scPRS-prioritized cell types aligned with the top-performing single-cell-type peak PRSs (Supplementary Fig. 3), providing additional insight into the rationale behind scPRS-based cell prioritization.

### scPRS reveals disease regulatory programs

**Fig. 4:**
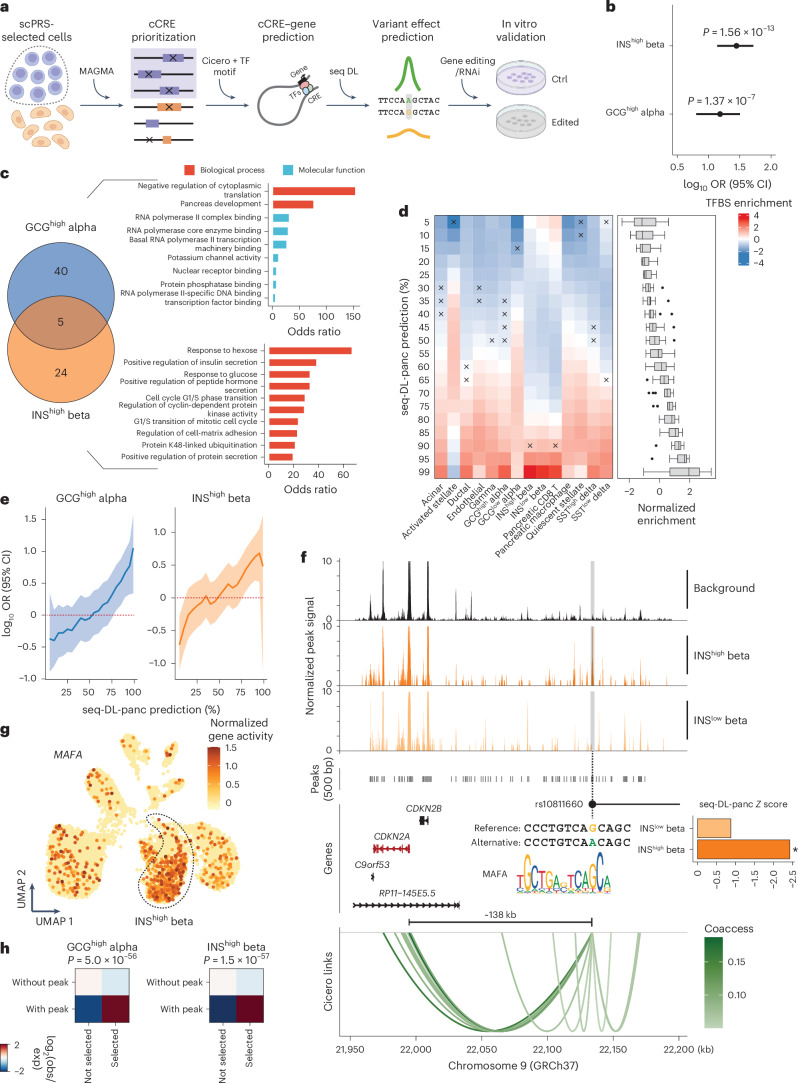
Cell-type-specific genetic regulation in T2D. **a**, Schematic of scPRS-based multiomic strategy for uncovering disease-relevant genetic regulation. RNAi, RNA interference. The schematic was created using BioRender.com. **b**, Enrichment of T2D-associated variants within cCREs that were differentially accessible in scPRS-prioritized cells. LD threshold *r*^2^ = 0.1 was used in clumping to retrieve an independent variant set (*n* = 783,082). *P* values were determined using a two-sided Fisher’s exact test. The log_10_(OR) and 95% CI are annotated by the dots and error bars, respectively. **c**, Candidate T2D genes and GO enrichment analysis results. Significant GO terms (adjusted *P* < 0.1, BH correction) with OR > 5 are visualized. **d**, Enrichment of TFBS-disrupting variants within seq-DL-panc-prioritized variants (various thresholds applied). seq-DL-panc, the sequence deep learning model trained on the pancreas snATAC-seq data. Enrichment was estimated by *t* statistics, where a total of 6,865,604 variants were tested. The box plot center line, limits and whiskers represent the median, quartiles and 1.5× the interquartile range (IQR), respectively. The dots indicate outliers falling above or below the end of the whiskers. Crosses indicate adjusted *P* > 0.1. **e**, Enrichment of seq-DL-panc-prioritized T2D-associated variants (various thresholds applied) within T2D-cCREs. ORs and CIs were determined using a two-sided Fisher’s exact test. The log_10_(OR) is annotated by the solid line and 95% CI is represented by the shaded area. The red dashed line indicates null enrichment. **f**, Illustration of the genetic regulation of rs10811660 in INS^high^ beta cells. In the bar plot, the asterisk indicates that the percentage of seq-DL-panc score is greater than 85%. In the gene plot, the mapped target gene is highlighted in red. In the link plot, links with coaccessibility > 0.05 are visualized; Coaccess, coaccessibility. **g**, The UMAP plot of the pancreas snATAC-seq dataset showing the expression of *MAFA* in individual cells. Gene expression was estimated on the basis of gene activity computed by Signac. INS^high^ beta cells are highlighted in the dashed closed curve. **h**, Ratio between observed and expected cell counts in GCG^high^ alpha (left) and INS^high^ beta (right) cells. *P* values were determined using a two-sided chi-square test.

As per model design, scPRS prioritizes cells that contain disease-associated variants within their differentially accessible chromatin regions. This feature empowers us to delve deeper into the regulatory circuits that contribute upstream of the disease across different cell types. To achieve this, we devised a layered multiomic strategy based on the trained scPRS model to systematically map cell-type-specific gene regulation underlying diseases (Fig. 4a and Methods).

For each disease-relevant cell type nominated by scPRS, we first identified the cCREs that were differentially accessible within scPRS-selected cells. Within these, we further prioritized cCREs (referred to as disease-relevant cCREs) that were significantly enriched with disease-associated variants using MAGMA^74^. To map cCRE–gene interactions, we performed coaccessibility analysis^75^ on the basis of the scATAC-seq data, supplemented by the closest-gene strategy given its effectiveness in nominating disease genes^76^. For each cell type, this procedure yielded a set of candidate disease genes associated with the disease-relevant cCREs.

To fine-map causal variants within disease-relevant cCREs, we used a sequence-based deep learning model^77–79^ that predicted chromatin accessibility across different cell types from the DNA sequence (Supplementary Fig. 4a and Methods). We trained the model using scATAC-seq data and then used it to predict the functional effects of individual variants on chromatin accessibility across cell types (Supplementary Fig. 7a and Methods). This completed the map of disease-relevant regulatory circuits composed of variant–cCRE–gene trios. Follow-up experiments were carried out in corresponding cell types to validate our predictions.

#### T2D

We first observed a significant enrichment of T2D-associated variants (GWAS *P* < 5 × 10^−8^) within differentially accessible cCREs for scPRS-prioritized cells (*P* < 1 × 10^−6^, two-sided Fisher’s exact test; Fig. 4b, Supplementary Fig. 7b and Methods). Using MAGMA, we identified 19 and 22 T2D-relevant cCREs (referred to as T2D-cCREs) in GCG^high^ alpha and INS^high^ beta cells, respectively (Supplementary Fig. 7c and Supplementary Table 1). Motif enrichment analysis for T2D-cCREs uncovered transcription factors (TFs) of functional importance in corresponding cell types (Supplementary Fig. 7c and Methods). For example, TEAD1 is a critical beta cell TF necessary for coordinating various aspects of adult beta cell function, including proliferative quiescence, mature identity and functional competence to uphold glucose homeostasis^80,81^. MAFB, whose motif is enriched in both cell types, is another pivotal TF in the islet. It is essential for the production and secretion of glucagon in alpha cells^82^ and for the maturation of beta cells^83^. A recent study demonstrated that XBP1 has a vital role in maintaining beta cell identity and repressing beta-to-alpha cell transdifferentiation, and is required for beta cell compensation and the prevention of diabetes in insulin resistance states^84^.

By mapping target genes of T2D-cCREs, we identified 45 and 29 candidate risk genes in GCG^high^ alpha and INS^high^ beta cells, respectively (Fig. 4c and Supplementary Table 1). The function of alpha cell genes was enriched with ‘pancreas development’ (GO:0031016) and ‘RNA polymerase core enzyme binding’ (GO:0043175) (adjusted *P* < 0.1, BH correction), whereas the function of beta cell genes was enriched with ‘response to hexose’ (GO:0009746), ‘positive regulation of insulin secretion’ (GO:0032024) and ‘response to glucose’ (GO:0009749) (adjusted *P* < 0.1, BH correction).

Trained on the pancreas snATAC-seq data, the sequence model exhibited high accuracy in peak prediction (AUROC, 0.819 ± 0.011; AUPRC, 0.639 ± 0.044; Supplementary Fig. 7d). We validated our variant effect prediction using two different approaches: expression quantitative trait locus (eQTL) analysis and TF-binding site (TFBS) prediction (Methods). Leveraging eQTL datasets generated in relevant tissues^85–88^, we observed that eQTLs tended to display larger effects on the basis of deep learning prediction in related cell types compared to non-eQTLs (Supplementary Fig. 7e). Additionally, variants with larger effects were more likely to alter TF binding^89^ (Fig. 4d). These results indicate that the sequence model had captured underlying gene regulation mechanisms. We also examined functional effects of T2D-associated variants (GWAS *P* < 0.05) located within T2D-cCREs in GCG^high^ alpha and INS^high^ beta cells (Methods). Variants with larger effect sizes showed higher enrichment in T2D-cCREs in corresponding cell types (Fig. 4e), providing additional support for the functional importance of T2D-cCREs we identified.

Combining multiomic evidence from eQTLs, TF binding and sequence model prediction fine-mapped T2D risk variants with functional implications (Supplementary Fig. 7f,g and Supplementary Table 1). One variant of particular interest is rs10811660, a T2D GWAS SNP^31^ (GWAS *P* = 1.30 × 10^−11^, *β* = −0.13, effect/alternative allele is A) residing within an INS^high^ beta cell-specific T2D-cCRE (chr9:22,133,835–22,134,336; *P* = 1.91 × 10^−14^, log_2_ fold change (FC) = 4.99; Fig. 4f). We predicted that the alternative allele specifically reduced the cCRE accessibility in INS^high^ beta cells (INS^high^ beta cell *Z* = −2.43, percentile = 96.84%; Fig. 4f). Furthermore, the affected cCRE was found to be coaccessible with *CDKN2A* (coaccessibility = 0.159; Fig. 4f). Previous studies demonstrated that the p16 inhibitor of cyclin-dependent kinase (p16^INK4A^), encoded by *CDKN2A*, restricts beta cell proliferation during aging, restricts beta cell regeneration, mediates overnutrition-related senescence and reduces insulin secretory function^90^. While rs10811660 has also been linked to a *CDKN2A* paralog, *CDKN2B*, because of their distance proximity^90^, our coaccessibility analysis suggested that this association might be a false-positive nomination (Fig. 4f). This conclusion was further supported by the islet eQTL data^88^, wherein rs10811660 was significantly associated with the expression of *CDKN2A* (*P* = 9.94 × 10^−4^, *Z* = 3.29) rather than that of *CDKN2B* (*P* > 0.05, *Z* = 0.40; Supplementary Fig. 7h). Additionally, we found that the alternative allele A disrupted the binding motif of MAFA (*P* < 1 × 10^−4^, motifbreakR^91^; Fig. 4f and Methods), a critical regulator of pancreatic beta cell function^92^, which was more highly expressed in beta cells (Fig. 4g). Collectively, our analysis suggests a genetic regulation influencing T2D risk; the T2D risk allele G (rs10811660) increases the abundance of MAFA binding, which further upregulates *CDKN2A* expression in INS^high^ beta cells. This aligns with previous evidence implicating that higher expression of *CDKN2A* may increase T2D risk^90^.

Lastly, we sought to characterize scPRS-selected cells beyond the resolution of predefined cell types. In particular, we compared selected cells to unselected ones from the same cell type. Differential accessibility analysis identified two peaks (chr10:94,479,864–94,480,365 and chr10:114,780,533–114,781,034) that were significantly enriched in scPRS-selected GCG^high^ alpha cells and three peaks (chr9:22,133,835–22,134,336, chr10:114,758,079–114,758,580 and chr10:114,780,533–114,781,034) enriched within INS^high^ beta cells. These marker peaks defined novel cell populations relevant to T2D (Supplementary Fig. 8), as informed by genetic risk. Consistent with this, we further classified each of these two cell types into subtypes on the basis of the accessibility of marker peaks and observed significant enrichment of scPRS-selected cells in the marker-defined subtype (*P* < 1 × 10^−50^, two-sided chi-square test; Fig. 4h). Of note, the peak chr10:114,780,533–114,781,034 was shared between the selected populations of alpha and beta cells; all marker peaks contained at least one T2D GWAS variant (Supplementary Table 1). The marker peaks of the selected GCG^high^ alpha cells were linked to genes such as *TCF7L2* and *CPEB3*, with *TCF7L2* also pinpointed in the selected INS^high^ beta cells, suggesting shared T2D biology across these two cell populations. The peak chr9:22,133,835–22,134,336 highlighted above (Fig. 4f) was one of the marker peaks of the selected INS^high^ beta cells; this peak contained the T2D risk SNP rs10811660, underscoring the cellular specificity of rs10811660 in impacting T2D risk.

#### HCM

We identified 137 and 358 HCM-relevant cCREs (referred to as HCM-cCREs) that were linked to 199 and 492 target genes in CDMs and pericytes, respectively (Supplementary Table 2). We observed only minimal overlap, with just one cCRE and 24 genes shared between these two cell types, highlighting their cell type specificity.

**Fig. 5:**
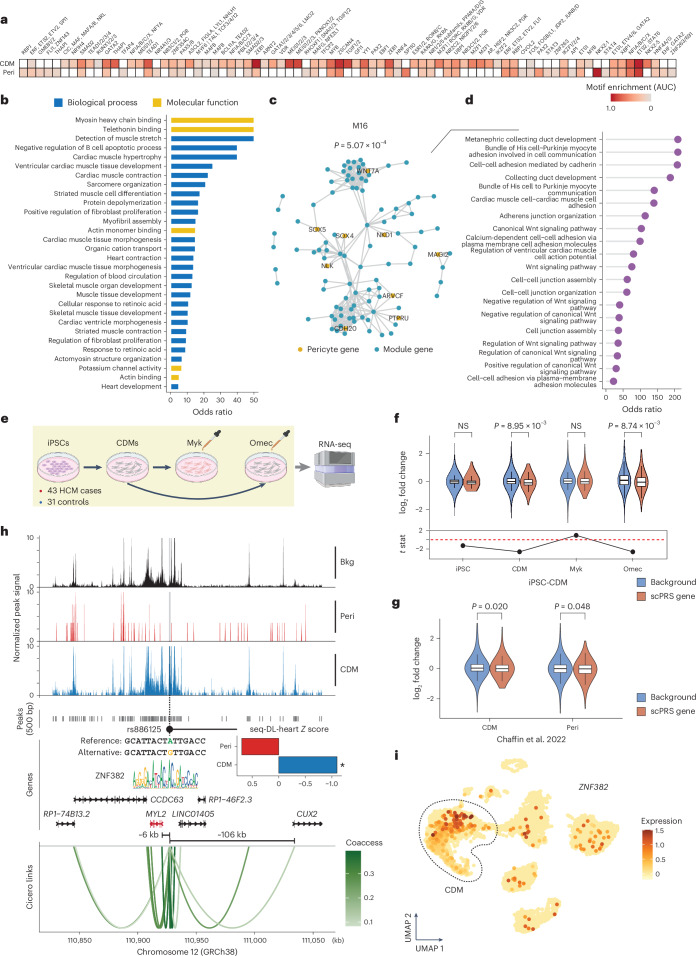
Cell-type-specific genetic regulation in HCM. **a**, Motif enrichment within HCM-cCREs identified in two HCM-relevant cell types including CDMs and pericyte. Motif enrichment was measured by AUC. Row-wise standardization was performed. Only significant enrichment (adjusted *P* < 0.1, Bonferroni correction) is colored. **b**, Bar plot of GO enrichment for CDM HCM risk genes. Significant GO terms (adjusted *P* < 0.1, BH correction) with OR > 5 are shown. **c**, The network module M16 enriched with pericyte HCM genes. *P* values were determined using a one-sided hypergeometric test. Edges between module genes are shown. **d**, Lollipop chart of GO enrichment (biological process) for M16 genes. Significant GO terms (adjusted *P* < 0.1, BH correction) are shown. **e**, Schematic of iPS cell RNA-seq experiments. Myk, mavacamten; Omec, omecamtiv mecarbil. The schematic was created using BioRender.com. **f**, Expression FC comparison between HCM risk genes and the background transcriptome in CDMs across different conditions. The box plot center line, limits and whiskers represent the median, quartiles and 1.5× the IQR, respectively. *P* values were determined using a two-sided *t*-test (*n* = 16,160). NS, not significant; stat, statistics. **g**, Expression FC comparison between HCM risk genes and the background transcriptome in HCM-relevant cell types based on an HCM snRNA-seq study. The box plot center line, limits and whiskers represent the median, quartiles and 1.5× the IQR, respectively. *P* values were determined using a two-sided *t*-test (*n* = 11,683). **h**, Illustration of the genetic regulation of rs886125 in CDMs. In the bar plot, the asterisk indicates a seq-DL-heart score percentage greater than 85%; seq-DL-heart, the sequence deep model trained on the left-ventricle snATAC-seq data. In the gene plot, differentially expressed target genes are mapped (in red). Bkg, background. **i**, The UMAP plot of the left-ventricle snRNA-seq dataset showing the expression of *ZNF382* in individual cells. Expression was estimated by normalized gene count. CDMs are highlighted in the dashed closed curve.

Our motif enrichment analysis for HCM-cCREs revealed TFs that have critical roles in corresponding cell types (Fig. 5a). For instance, TEAD1 is a pivotal regulator involved in maintaining the proper functioning of adult CDMs, whose loss of function has been associated with dilated cardiomyopathy^93^. GATA4 exerts notable control over cardiac gene expression, impacting embryonic development, CDM differentiation and stress responsiveness of the adult heart^94^. NKX2-5 is a central regulator of heart development and pathogenic mutations within it contribute to progressive cardiomyopathy and conduction defects^95^. Additionally, RBPJ inactivation has been linked to the development of disease-promoting properties in brain pericytes^96^. STAT3 serves as a key regulator of cell–cell communication within the heart, a critical aspect of pericyte functionality^97^.

HCM risk genes identified in CDMs exhibited functional importance in CDMs and cardiomyopathy, such as ‘myosin heavy chain binding’ (GO:0032036), ‘cardiac muscle contraction’ (GO:0060048) and ‘sarcomere organization’ (GO:0045214) (adjusted *P* < 0.1, BH correction; Fig. 5b). No Gene Ontology (GO) enrichment was observed for pericyte genes, suggesting a marked functional diversity within this gene set. To better dissect this heterogeneity, we carried out a network analysis on the basis of the protein–protein interactions (PPIs)^98^ (Methods), in which one module M16 was significantly enriched with HCM pericyte genes (*P* = 5.07 × 10^−4^, hypergeometric test; adjusted *P* = 0.034, BH correction; Fig. 5c). Genes within this module displayed GO enrichment in various pericyte functions, such as ‘cell–cell adhesion mediated by cadherin’ (GO:0044331), ‘cell–cell junction assembly’ (GO:0007043) and ‘cadherin binding’ (GO:0045296) (adjusted *P* < 0.1, BH correction; Fig. 5d and Supplementary Fig. 9a).

To better understand the gene function in the disease context, we analyzed an RNA sequencing (RNA-seq) dataset^27^ of induced pluripotent stem cell (iPS cell)-derived CDMs obtained from 43 HCM cases and 31 healthy controls (Fig. 5e). Bulk RNA-seq profiling was conducted under four conditions: iPS cells, differentiated CDMs, mavacamten-treated^99^ (an HCM drug recently approved by FDA) CDMs and omecamtiv mecarbil^100^ (a heart failure drug serving as the negative control) treated CDMs. Notably, although the CDM HCM genes exhibited no expression difference in iPS cells between HCM cases and healthy controls, their expression was significantly reduced in differentiated HCM CDMs compared to control cells (*P* = 8.95 × 10^−3^, two-sided *t*-test; Fig. 5f, Supplementary Table 3 and Methods). Intriguingly, the downregulation of HCM genes was rescued by mavacamten treatment (*P* = 0.017, two-sided *t*-test) but persisted in omecamtiv mecarbil treatment (*P* > 0.05, two-sided *t*-test; Fig. 5f). The reduced expression of HCM genes identified in CDMs and pericytes was also confirmed in corresponding cell types using an independent HCM single-cell transcriptome dataset^101^ (CDM *P* = 0.02, pericyte *P* = 0.048, two-sided *t*-test; Fig. 5g), while showing cell type specificity (Supplementary Fig. 9b,c). These results demonstrate the disease relevance of our HCM genes.

We trained a different sequence deep learning model on the basis of the snATAC-seq dataset of the left ventricle (AUROC, 0.846 ± 0.019; AUPRC, 0.658 ± 0.032; Supplementary Fig. 9d). Variant effects predicted by the model agreed well with eQTL profiling^85,102^ and TFBS prediction (Supplementary Fig. 9e,f). HCM-cCREs presented increased enrichment of HCM-associated variants (GWAS *P* < 0.05) with larger effects (Supplementary Fig. 9g).

The sequence deep learning prediction, together with eQTL and TFBS analyses, fine-mapped novel cell-type-specific HCM risk variants (Supplementary Fig. 9h,i and Supplementary Table 2). As an example, the CDM-specific HCM-cCRE (chr12:110,927,025–110,927,526; *P* = 2.5 × 10^−3^, log_2_ FC = 1.94; Fig. 5h) contained a nominally significant GWAS^30^ variant rs886125 (GWAS *P* = 0.019, *β* = −0.149, effect/alternative allele = G) and was coaccessible (coaccessibility = 0.367) with *MYL2*, a widely recognized HCM gene^80^. On the basis of our predictions, the alternative allele G specifically decreased the cCRE within CDMs (CDM *Z* = −1.10, percentile = 87.62%; Fig. 5h) and it disrupted the TFBS of ZNF382 (*P* < 1 × 10^−4^, motifbreakR; Fig. 5h), which is known as a transcriptional repressor^103^. These results together suggest that the risk-increasing allele A, bound by ZNF382, would lower the expression of *MYL2* in CDMs. This was supported by the eQTL data^86^ in which the risk allele A was associated with decreased expression of *MYL2* (*P* = 0.011, *β* = 0.125; GTEx artery aorta). Additionally, using our paired snRNA-seq data, we found that *ZNF382* was more highly expressed in CDMs (Fig. 5i), highlighting its cell-type-specific role in gene regulation.

#### AD

**Fig. 6:**
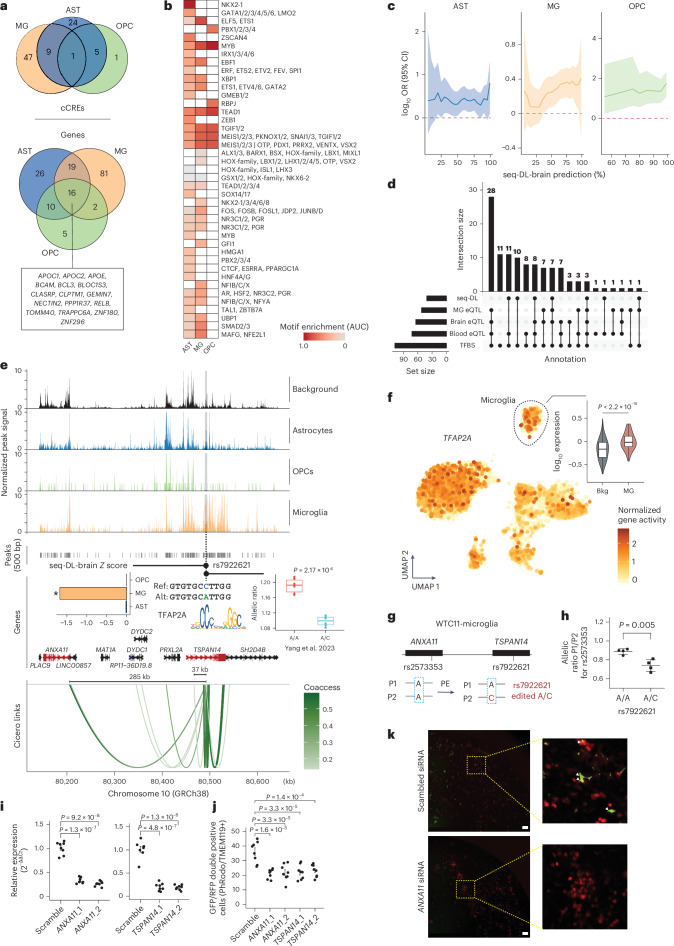
Cell-type-specific genetic regulation in AD. **a**, Venn diagram of AD-relevant cCREs (top) and genes (bottom) identified by the scPRS-based multiomic strategy. AST, astrocyte; MG, microglia. **b**, Motif enrichment within AD-cCREs across different cell types. Motif enrichment was measured by AUC. Column-wise standardization was performed. Only significant enrichment (adjusted *P* < 0.1, Bonferroni correction) is colored. *P* values were determined using a hypergeometric test. **c**, Enrichment of seq-DL-prioritized AD-associated variants (various thresholds applied) within AD-cCREs. ORs and CIs were determined using a two-sided Fisher’s exact test. The log_10_(OR) is annotated by the solid line and the 95% CI is represented by the shaded area. The red dashed line indicates null enrichment. **d**, Summary statistics of fine-mapped AD risk variants in microglia using different annotations. **e**, Illustration of the genetic regulation of rs7922621 in microglia. Box plot: the box plot center line, limits, and whiskers represent the median, quartiles and 1.5× the IQR, respectively. *P* values were determined using a two-sided *t*-test. ref, reference; alt, alternative. In the bar plot, the asterisk indicates a seq-DL-brain score percentage greater than 85%; seq-DL-brain, the sequence deep model trained on the cortex scATAC-seq data. In the gene plot, differentially expressed target genes are mapped (in red). In the link plot, links with coaccessibility > 0.05 are shown. Coaccess, coaccessibility. **f**, The UMAP plot of the cortex scATAC-seq dataset showing the expression of *TFAP2A* in individual cells. In the violin plot, *P* values were determined using a two-sided *t*-test. Gene expression was estimated on the basis of the gene activity computed by Signac. Microglia are highlighted in the dashed closed curve. **g**, Diagram showing the haplotypes of variants in wild-type and rs7922621 prime-edited WTC11-derived microglia. The P1 allele has the risk allele (A), while the P2 allele has the nonrisk allele (C). PE, prime editing. **h**, Allelic imbalance between P1 and P2 alleles for *ANXA11* quantified by rs2573353 in rs7922621 wild-type (A/A) and prime-edited (A/C) WTC-derived microglia (*n* = 4 replicates). The center line and error bar represent the mean and s.d, respectively. *P* values were determined using a two-sided *t*-test. **i**, RT–qPCR quantification of relative mRNA levels in iMGs treated with siRNAs targeting AD genes or scrambled siRNA (*n* = 2 siRNAs for each gene; *n* = 8 replicates for each condition). mRNA levels were normalized to GAPDH. *P* values were determined using a two-sided *t*-test. Data are presented as the mean ± standard error. **j**, Quantification of the number of TMEM119^+^ cells colocalized with pHrodo particles indicating phagocytosed beads (*n* = 2 siRNAs for each gene; *n* = 8 replicates for each condition). A one-way ANOVA with Tukey’s HSD test was used for comparison between siRNA targeting AD genes and scrambled siRNA. Data are presented as the mean ± standard error. **k**, Representative images of TMEM119^+^ (red) iMGs treated with *ANXA11* siRNA or scrambled siRNA showing colocalization of phagocytosed pHrodo particles (green, highlighted with arrows). Images were captured 2 h after incubation with pHrodo. Parts of the images are zoomed in for better visualization. Scale bar, 100 μm.

We first confirmed a significant enrichment of AD-associated variants (GWAS *P* < 5 × 10^−8^) within differentially accessible cCREs in scPRS-prioritized cells (*P* < 5 × 10^−3^, two-sided Fisher’s exact test; Supplementary Fig. 10a). We identified 39, 57 and 6 AD-relevant cCREs (referred to as AD-cCREs) that were linked to 71, 118 and 33 target genes in astrocytes, microglia and OPCs, respectively (Fig. 6a and Supplementary Table 4). Numerous AD-cCREs and genes were shared across different cell types, among which we recognized multiple well-established AD genes, such as the *APOE* region genes (*BCAM*, *NECTIN2*, *TOMM40*, *APOE* and *APOC1*), *BCL3* and *PPP1R37*. This signifies their versatile roles in AD pathogenesis.

Next, we examined the function of AD-cCREs and candidate genes in corresponding cell types. We found that AD-cCREs were enriched with binding motifs of cell-type-critical TFs (Fig. 6b). For example, astrocyte AD-cCREs displayed exclusive motif enrichment for GATA4, a regulator of astrocyte cell proliferation and apoptosis^104^; microglia AD-cCREs exhibited significant motif enrichment for SMAD3, which cooperates with PU.1 to enable transcription of some microglia-specific genes^105^; OPC AD-cCREs were exclusively enriched with the RBPJ motif, which is a repressor of OLIG2, a major determinant of oligodendrocyte differentiation and myelination^106^. Additionally, the AD candidate genes also presented cell-type-specific functions. For instance, astrocyte AD genes were enriched with the function of ‘regulation of complement activation, classical pathway’ (GO:0030450), microglia AD genes displayed enrichment in ‘negative regulation of endocytosis’ (GO:0045806) and OPC AD genes exhibited significant enrichment in ‘IκB kinase and NF-κB signaling’ (GO:0007249) (adjusted *P* < 0.1, BH correction; Supplementary Table 5).

To characterize the variant effect within AD-cCREs, we trained a sequence deep learning model on the basis of the cortex scATAC-seq data (AUROC, 0.916 ± 0.017; AUPRC, 0.795 ± 0.059; Supplementary Fig. 10b). We confirmed the agreement in variant effect prediction between the sequence model and two other approaches, including QTL (expression and chromatin accessibility) analysis and TFBS prediction (Supplementary Fig. 10c,d). We also uncovered an enrichment of large-effect AD-associated variants (GWAS *P* < 0.05) within AD-cCREs across all three relevant cell types, where the enrichment was positively correlated with variant effect (Fig. 6c).

We fine-mapped AD risk variants by combining multiomic evidence (Fig. 6d, Supplementary Fig. 10e,f and Supplementary Table 4). Among the prioritized variants, we recognized numerous cell-type-specific risk loci that were previously reported in the literature. For example, the AD risk variant rs10792832 (GWAS^29^ *P* = 7.56 × 10^−16^, *β* = −0.12, effect allele/reference = A) was associated with the deactivation of a microglia-specific cCRE for *PICALM*^60^, aligning with our prediction (microglia *Z* = −1.98, *PICALM* coaccessibility = 0.246). Another AD risk variant rs13025717 (GWAS *P* = 2.98 × 10^−15^, *β* = 0.13, effect/alternative allele = T), which represses a microglia cCRE for *BIN1* (ref. ^35^), was also prioritized by our analysis (microglia *Z* = −2.60, *BIN1* coaccessibility = 0.382). A recent study validated the role of rs1532278 (GWAS *P* = 3.27 × 10^−16^, *β* = −0.13, effect/reference allele = T) in modulating *CLU* expression in astrocytes^107^, supporting our findings (astrocyte *Z* = −0.498, *CLU* coaccessibility = 0.356;).

In addition to known AD risk loci and genes, our analysis discovered novel genetic factors. One of particular interest is rs7922621, which is nominally significant across the genome^29^ (GWAS *P* = 2.78 × 10^−5^, *β* = 0.08, effect/alternative allele = A). This variant resides within a microglia-specific AD-cCRE (chr10:82,251,479–82,251,979; *P* = 1.99 × 10^−19^, log_2_ FC = 2.39; Fig. 6e). According to the sequence model prediction, rs7922621 diminished the accessibility of this cCRE exclusively in microglia but not in other cell types (microglia *Z* = −1.68, percentile = 96.63%; Fig. 6e). Coaccessibility analysis further predicted that this cCRE regulated the expression of two genes: *ANXA11* and *TSPAN14* (Fig. 6e). Importantly, a recent study reported a reduction in local chromatin accessibility associated with rs7922621 in human PS cell-derived microglia^108^. They further validated the reduced expression of *TSPAN14* caused by rs7922621 using prime editing (*P* = 2.17 × 10^−6^, two-sided *t*-test; Fig. 6e). Of note, another variant, rs7910643, located within the same cCRE and in strong LD with rs7922621 (*r*^2^ = 1.0, estimated in the 1,000 Genomes EUR population), was shown to be nonfunctional^108^, consistent with our prediction (microglia *Z* = 0.29, percentile < 85%; Supplementary Table 4).

To further elucidate the regulatory program involving rs7922621, we conducted TF motif analysis and identified one TF, TFAP2A, whose binding site was disrupted by rs7922621 (*P* < 1 × 10^−4^, motifbreakR; Fig. 6e). The TFAP2 family is known for its pivotal role in regulating both embryonic and oncogenic development^109^. Furthermore, *TFAP2A* expression showed a significant elevation in microglia compared to other cell types (*P* < 2.2 × 10^−16^, two-sided *t*-test; Fig. 6f), suggesting its functional importance in microglia, although further evidence is required to validate these conclusions.

### Prime editing of rs7922621 alters expression of both *ANXA11* and *TSPAN14* in microglia

Our scPRS-based analysis pinpointed rs7922621 (chr10:82,251,544:C>A) as a candidate AD risk variant and predicted that it regulates two genes (*ANXA11* and *TSPAN14*) by altering the accessibility of a microglia-specific cCRE (chr10:82,251,479–82,251,979; Fig. 6e). Our prior study^108^ validated the association between rs7922621 and this cCRE and further demonstrated that the prime editing of rs7922621, converting the risk allele (A) to the nonrisk allele (C) in WTC11 (A/A to A/C)-derived microglia (a male iPS cell line), led to an increase in *TSPAN14* expression. Leveraging the rs7922621-edited clones^108^, we further examined its regulatory role on *ANXA11* (Fig. 6g and Methods). We observed a similar trend in the allelic expression changes of *ANXA11* associated with rs7922621 in WTC11-derived microglia, with the edited nonrisk allele upregulating *ANXA11* compared to the risk allele (*P* = 0.005, two-sided *t*-test; Fig. 6h). We note that, in contrast to *TSPAN14*, *ANXA11* exhibits a long-range interaction (~285 kb) with rs7922621 (Fig. 6e). Altogether, these results suggest an upstream role of rs7922621 in modulating the expression of both *ANXA11* and *TSPAN14* in microglia, with the AD risk allele (A) reducing their expression.

### Suppression of *ANXA11* and *TSPAN14* impairs microglial phagocytosis

To elucidate the function of *ANXA11* and *TSPAN14* in microglia, we examined the effect of knockdown of these genes on microglial phagocytic activity. In particular, we individually suppressed *ANXA11* and *TSPAN14* in iPS cell-derived microglia-like cells^110,111^ (iMGs) using small interfering RNA (siRNA), in which two different siRNAs were tested for each gene. Phagocytosis activity was measured using a fluorescent readout of pHrodo particles. A reduction in expression following siRNA treatment was confirmed for both genes (*P* < 1 × 10^−4^, two-sided *t*-test; Fig. 6i). Notably, suppression of these two genes resulted in significantly decreased iMG uptake of pHrodo particles compared to scrambled siRNA treatments (adjusted *P* < 1 × 10^−3^, one-way analysis of variance (ANOVA) with Tukey’s honestly significant difference (HSD) test; Fig. 6j). These results were consistent across treatments using different siRNAs. Our experimental results validated the functional importance of *ANXA11* and *TSPAN14*, showing that their suppression impaired microglial phagocytosis, thus supporting the pivotal role of rs7922621 in modulating AD risk through its impact on microglial function.

## Discussion

GWAS has substantially advanced our understanding of the genetic basis of complex human diseases^112^. Traditionally, these studies aim to identify genetic loci that reach genome-wide significance (that is, GWAS *P* < 5 × 10^−8^). However, for many diseases, the best predictive performance is only achieved by including nominally significant or even nonsignificant variants in PRS calculation^113^. This suggests that the genetic factors contributing to diseases extend beyond those genome-wide significant loci and cannot be fully uncovered by conventional approaches^114^. While scientists have been calling for larger GWAS consortia and meta-analyses to identify more disease risk loci^115^, it remains an open question how to increase the discovery power given relatively limited sample size. Incorporating prior knowledge or multiomic data into genetic association analysis has proven to be an effective solution^67,116^.

PRS has been demonstrated as a powerful tool to predict an individual’s disease risk. However, it lacks the ability to provide insights into disease mechanisms. From the perspective of modern machine learning, model interpretation is critical in uncovering latent features that contribute to prediction and understanding how models make decisions^117^. As a score computed by aggregating a wide range of variants, PRS offers limited knowledge on the significance of each variant in prediction. Moreover, distinguishing causal variants from statistically correlated elements poses an even greater challenge. For example, a variant can be associated with the disease through its linkage with the causal variant, yet both are treated equivalently within a PRS model. This lack in biology-informed model interpretability can, in turn, constrain predictive performance such as generalizability^15^.

We designed scPRS, a deep learning-based PRS framework, to address these challenges. scPRS leverages single-cell epigenetic data to dissect the genome-wide PRS and then integrates single-cell-level PRSs using a GNN. By breaking down PRS into higher-resolution components informed by cellular functions, scPRS not only enhances its predictive power but also allows for a systematic exploration of cellular and molecular basis for diseases. Applications to various diseases have shown that scPRS outperformed a variety of existing PRS methods. Importantly, this superior predictive performance of scPRS was achieved using less than 11% of all the variants (that is, variants located within open chromatin regions; Supplementary Table 6), highlighting the importance of incorporating functional data^15^ and suggesting a notable contribution of noncoding variants to disease risk^118^.

We showcased the effectiveness of scPRS in identifying disease-critical cells. Our method is not confined to cell clustering and predefined cell types, offering an unbiased, agnostic analysis. Through single-cell-resolved modeling, scPRS can discover disease-relevant cell populations by integrating genetic insights. This was demonstrated in identifying previously uncharacterized T2D-related alpha and beta cell populations defined by scPRS-selected cells. Similar analysis was performed for other three diseases but no significance was observed. Unlike the selected cells enriched within highlighted cell types, the model-prioritized cells in other cell types were sparsely distributed in the epigenome space, suggesting less homogeneous cellular functions. The selection of these cells was likely because of the randomness of model initialization and training, as well as the technical noise inherent in single-cell sequencing. Therefore, we recommend considering these cells as background.

The cell type prioritization results agreed well between scPRS and sLDSC but the most notable difference occurred for HCM, where sLDSC failed to identify any relevant cell types. This lack in power could be explained by the difference between bulk and individual-level or single-cell-level modeling. First, sLDSC estimates heritability enrichment across an ensemble of open chromatin regions within a specific cell type but this bulk-level approach does not account for the variation among individual cells. This limitation can lead to confounding by non-disease-relevant regions, reducing its ability to identify disease-critical cells with a high sensitivity. In contrast, scPRS weighs the importance of each cell relative to others, providing a global model that captures cross-cell variation in heritability enrichment. The subsequent cell enrichment analysis within each cell type enables an effective disease–cell association discovery. Another advantage of this single-cell-resolved approach is its ability to identify novel disease-relevant cell populations that are not annotated in the single-cell dataset, which has been demonstrated for T2D. Second, sLDSC works in the GWAS space and its performance can be influenced by the power of the original GWAS. This may explain why sLDSC identified zero relevant cell types for HCM, as the HCM GWAS was relatively underpowered, with only two genome-wide significant variants^30^ (*P* < 5 × 10^−8^). Although GWAS summary statistics are part of the input to scPRS, it selects cells whose cell-level PRSs best differentiate individual patients from controls rather than relying on the overall GWAS performance. This individual-level modeling further increases the power to identify disease-relevant cells. Of note, the cell type enrichment *P* values for sLDSC, SCAVENGE and scPRS are not directly comparable. Significant cell types were identified by comparing *P* values derived from the same method. Hence, the conclusions regarding cell type enrichment are comparable across different approaches.

Several recent studies^46,119,120^ have also achieved prioritization of disease-relevant cells at the single-cell level. However, these approaches rely on GWAS summary statistics and, thus, lack predictive power. Moreover, superior to these methods, scPRS enables pinpointing disease risk variants, genes and regulatory programs across different cellular contexts, substantially enhancing the power and resolution of genetic discovery. This advancement is exemplified by rs7922621, which was pinpointed by scPRS-based analysis as a candidate AD risk variant but missed by GWAS because of its nominal significance. rs7922621 was also nominated in two recent studies^108,121^, where it was mapped to *TSPAN14* in microglia as the target gene. Our scPRS-based analysis further linked rs7922621 to another gene *ANXA11*. The understanding of the role of *ANXA11* in neurodegenerative disease is rapidly evolving; it was first implicated as a genetic cause of amyotrophic lateral sclerosis^122^ (ALS) and later corticobasal syndrome^123^. Recently, ANXA11 was also revealed as a central pathology in specific subtypes of frontotemporal dementia^124^. It is interesting that ANXA11 pathology extends beyond neurons; for example, muscle pathology has been observed as part of a multisystem proteinopathy with prominent myopathy^125^. A gap remains in our understanding of the biology underlying *ANXA11* dysfunction. ANXA11 protein is involved in the tethering of RNA granules, including lysosomes with a role in RNA transport^126^. However, this mechanism does not easily account for all of the pathological observations made. Our results suggest a role of *ANXA11* in microglia that underpins AD risk. This is reminiscent of observations of *TBK1*, another ALS gene where distinct pathological mechanisms have been observed in neurons and microglia^127^. We experimentally validated the regulatory relationship between rs7922621 and *ANXA11* and the function of these two genes (*ANXA11* and *TSPAN14*) in maintaining microglial phagocytosis. Our data support a model where rs7922621 increases AD risk by reducing a microglia cCRE targeting *ANXA11* and *TSPAN14* and then suppressing their expression, which impairs microglial phagocytosis.

It is worth noting that we identified rs7922621 in microglia by starting with a comparison analysis of cells prioritized by scPRS, without which the subsequent analysis would not have been possible (Fig. 4a). While microglia are a well-recognized cell type in AD, we pinpointed this AD-microglia linkage without incorporating any prior knowledge, using an agnostic, unbiased approach. This serves as a positive control to demonstrate the effectiveness of scPRS in identifying disease-critical cells. The nomination of new disease-relevant cell populations that are not annotated in the original single-cell dataset (Fig. 4h) underscores the discovery power of scPRS beyond simply reaffirming known disease–cell associations.

HCM is a genetic condition with a heritability of up to 50% in its familial form^53^ and an estimated SNP-based heritability ranging from 0.17 to 0.29 (ref. ^128^). The genetic study of HCM has been traditionally focused on rare pathogenic coding variants^53^. However, approximately 40% of persons with HCM remained unexplained by known pathogenic variants. Previous HCM GWASs for common variants have been underpowered, likely because of the limited number of participants recruited, resulting in an incomplete knowledge of the genetic architecture^129^. Our scPRS-based analysis greatly expands our understanding of HCM genetics, highlighting the critical role of common noncoding variants in influencing disease risk. Our findings underscore the importance of regulatory variants that have been largely overlooked in the HCM field. These variants may act as risk modifiers through modulating the expression of their target genes, including known HCM risk genes such as *MYL2*. Although further validations are necessary, our results shed light on the complexity of HCM genetics and biology.

We also constructed C+T PRSs using disease-associated variants (GWAS *P* < 0.05) located within disease-relevant cCREs. Disease cCREs from different prioritized cell types were aggregated. We observed that these selected variants dominated scPRS prediction, showing comparable performance for HCM and T2D (Supplementary Fig. 11). This result supports the effectiveness of our scPRS-based framework in identifying cell-type-specific disease-related variants. However, we caution that it cannot be concluded that this PRS, built on selected variants, is comparable to scPRS in terms of prediction, as it was derived from scPRS after explicitly seeing all samples, which may have caused the overfitting issue.

Single-cell genetics is an emerging field that is reshaping our understanding of genotype–phenotype relationships^17^. By integrating single-cell genomic data into genetic analysis, single-cell genetics provides a novel instrument to link genetic variants to diverse cellular processes. This is well exemplified by single-cell eQTL studies^130–132^, which enable the identification of context-dependent eQTLs that vary across cell states or cell types. scPRS lays the methodological foundation of single-cell genetics, marking a step toward mapping the genetic basis of complex diseases in a single-cell-resolved context.

We note that scATAC-seq only annotates genomic regions that are potentially involved in transcriptional regulation (for example, promoters, enhancers and silencers), whereas other layers of functions, such as proteins, translation and post-transcription, are not considered in our current modeling. Considering the heterogeneity and complexity of a disease’s genetic architecture, the prediction of scPRS could be suboptimal for certain diseases wherein coding, splicing or other variants have an important role. Indeed, we observed better predictive performance of C+T over scPRS for T2D. As a compensation, we further incorporated nonpeak PRSs constructed using variants located outside scATAC-seq peaks into scPRS, resulting in scPRS+, which outperformed all baseline PRS methods across the board. Following the same design principle, scPRS can be extended to include a wider range of variants by integrating additional modalities, such as scRNA-seq^133,134^ and single-cell DNA methylation^135,136^. This will be explored in our future work.

Considering both time and space complexities, we recommend starting with a moderately large number of cells, such as the 10,000 used in this manuscript, when applying scPRS in practice. This approach ensures coverage of cases where the disease or phenotype is driven by rare cell types or populations. Moreover, this strategy aligns with the exploratory nature of scPRS, as it is a discovery process in which the disease-relevant cells are largely unknown until analysis, typically requiring multiple iterations of testing.

In summary, scPRS stands as a versatile framework for simultaneous disease prediction and biological discovery, enabling the dissection of the genetic, cellular and molecular heterogeneity underlying complex diseases.

## Methods

### Single-cell multiome dataset

Single-cell multiome (snRNA-seq + snATAC-seq) data of the human left ventricle and lung were processed and clustered on the basis of RNA modality using Scanpy^137^. The cells with high-quality RNA information (total detected gene > 500, total unique molecular identifiers < 20,000 and mitochondrial read percentage < 10%) were selected for further analysis. Doublets were filtered using scrublet^138^ with parameters min_counts = 1, min_cells = 10, min_gene_variability_pctl = 90 and n_prin_comps = 30. The thresholds for doublet removal were decided per sample on the basis of the distribution of doublet scores in real versus simulated cells. The top 3,000 highly variable genes were selected by combining the results from each sample separately with seurat_v3 mode. The cell-by-gene count matrices were normalized and scaled. ALLCools with a Python implementation of Seurat integration was used for correction of batch effect between samples with 50 PCs and 30 canonical correlation dimensions^136,139^. Leiden clustering was performed on a *k*-nearest neighbor (kNN; *k* = 25) graph. The cell clusters were annotated and merged to cell types by comparing the expression level of predefined marker genes across clusters. The marker genes in Litviňuková et al. (2020) and Tucker et al. (2020)^140,141^ were used to annotate the heart cell types.

We also examined the ATAC modality of these cells following the methods described below to ensure that these cells also have high-quality open chromatin information. The cells that did not pass ATAC quality controls (QCs) or constituted an ambiguous cluster in ATAC cell embedding were removed, resulting in 10,233 and 10,330 cells retained for downstream analysis for HCM and severe COVID-19, respectively.

### scATAC-seq datasets

The cell type labels for the human pancreas and cortex in the original datasets^33,35^ were used. To generate cell embeddings, scATAC-seq data were processed and clustered using snapATAC2 (ref. ^142^) and ALLCools^136,139^. The fragment files were processed to generate cell-by-bin matrices at 5-kb resolution using snapATAC2 (ref. ^142^). The cells with 2,000–50,000 total reads and transcription start site (TSS) enrichment > 5 or 7 according to the distribution in specific samples were retained. The cell embeddings were computed with latent semantic indexing (LSI) and batch effects were corrected using the canonical correlation analysis (CCA) LSI mode in ALLCools. Cell-by-peak matrices at 500-bp resolution were generated by calling peaks per cell cluster using snapATAC2. For cortex data, superior and middle temporal gyri and middle frontal gyrus samples were used for AD analysis, resulting in 11,738 cells. For pancreas data, we randomly sampled 10,000 of 64,948 cells covering all annotated cell types for computational acceleration. The single-cell data^121^ we used in the replication experiments were processed and QCed similarly.

### Cell–cell similarity network

Following a previous study^46^, we used the mutual kNN (M-kNN) to measure the similarity between two different cells. We first used LSI to extract low-dimensional embeddings for individual cells. For cortex and left-ventricle datasets encompassing multiple samples, batch effects were corrected using both CCA and Harmony^143^ and integrated latent embeddings were adopted. Next, we computed the Euclidean distance for pairs of cells using their embeddings and then constructed the kNN graph $\hat{G}\in {{\mathfrak{R}}}^{M\times M}$ on the basis of this distance matrix, in which we defined ${\hat{G}}_{i,\;j}=1(i,j=1,\ldots ,M)$ if cell *j* is within the top *k* closest cells of cell *i* and ${\hat{G}}_{i,\;j}=0$ otherwise. The M-kNN graph *G* was then defined as the graph whose edges connect nodes (that is, cells) that are mutually kNNs of each other, which was calculated by $G=\hat{G}\circ {\hat{G}}^{T}$, where $\circ$ denotes the element-wise multiplication.

### Target cohorts for T2D and AD

T2D and AD target cohorts were constructed on the basis of the UKBB. All the disease cases were defined according to the ICD-10 (tenth revision of the International Statistical Classification of Diseases and Related Health Problems) code. In particular, all Caucasian individuals with a disease ICD-10 code in the inpatient record, death record or diagnosis summary record were defined as the disease participants. We used E11.9 and G30.9 for AD and T2D, respectively. This resulted in 1,096 T2D and 932 AD cases. We randomly sampled an equal number of healthy controls by matching sex, age and ancestry information for each case group. In addition, individuals with a similar or related phenotype with the disease (T2D: E10, E11, E12, E13, E14, E23.2, N08.3, N25.1, O24, P70.2, Z13.1, Z83.3 and R73.9; AD: F00, G30, F01, F02, F03 and F05) were excluded from constructing the control group. In this study, overweight individuals (body mass index (BMI) ≥ 25) were excluded from constructing the T2D cohort. BMI for each individual was defined as the mean of four BMI measurements in the UKBB Data Field 21001.

### Target cohort for HCM

The recruitment of the HCM cohort was part of our California Institute for Regenerative Medicine (CIRM) cardiomyopathy project^27^. The targeted population constituted persons with various cardiac procedures and noncardiac participants with genetic conditions in clinic who were identified to us by their clinical providers. Noncardiac participants were recruited in person during onsite clinic days or over the phone with permission by the providers. Healthy volunteers were recruited from our cardiovascular prevention clinic (that is, persons with no diagnosis of heart disease).

Library preparation and sequencing was performed by Macrogene (first ten samples) and Novogene on genomic DNA we extracted from iPS cells (Qiagen DNeasy kit). Paired-end 150-bp reads were acquired on the Illumina HiSeq X Ten for a minimum of 90 Gb of data. Reads were processed using Sentieon’s FASTQ-to-VCF pipeline (Sentieon version 201808.07)^144^. This pipeline is a drop-in replacement for a Burrows–Wheeler aligner (BWA)^145^ plus GATK best-practices^146^ pipeline for germline single-nucleotide variations (SNVs) and indels but has been highly tuned for optimal computational efficiency. BWA alignment to hg38 was followed by deduplication, realignment, base quality score recalibration and variant calling to generate g.vcf files for each sample. Coverage was assessed (GATK version 3.7)^27^. Individual sample g.vcf files were joined and variant quality score recalibration was performed.

### Target cohort for severe COVID-19

The VA COVID-19 cohort was derived from the VA MVP. The VA MVP is an ongoing national voluntary research program that aims to better understand how genetic, lifestyle and environmental factors influence veteran health^28^. Briefly, individuals aged 18 to over 100 years old have been recruited from over 60 VA medical centers nationwide since 2011 with current enrollment at >800,000. Informed consent is obtained from all participants to provide blood for genomic analysis and access to their full electronic health record data within the VA before and after enrollment. The study received ethical and study protocol approval from the VA central institutional review board (IRB) in accordance with the principles outlined in the Declaration of Helsinki. COVID-19 cases were identified using an algorithm developed by the VA COVID national surveillance tool based on reverse transcription (RT)–qPCR laboratory test results conducted at VA clinics, supplemented with natural language processing on clinical documents for SARS-CoV-2 tests conducted outside of the VA^147^. This resulted in the VA COVID-19 WGS cohort of 2,716 persons with COVID-19 spanning a wide range of ages and ancestries. We defined severe COVID-19 cases as persons who were hospitalized, received acute care, stayed in the intensive cure unit or were deceased and controls as those who did not meet these criteria. To minimize potential confounders, we restricted our analysis to nonelderly individuals (age < 65).

DNA isolated from peripheral blood samples was used for WGS. Libraries were prepared using KAPA hyper prep kits, PCR-free according to manufacturers’ recommendations. Sequencing was performed using an Illumina NovaSeq 6000 System (Illumina) with paired-end 2× 150-bp read lengths and Illumina’s proprietary reversible terminator-based method. The specimens were sequenced to a minimum depth of 25× per specimen and an average coverage of 30× per plate.

### Independent target cohorts

The GoT2D cohort including 2,874 individuals was used as the independent target cohort for T2D. Samples were sequenced using three technologies: deep whole-exome sequencing, low-pass (4×) WGS and OMNI 2.5M genotyping. Genotypes (SNVs, indels and structural variants) were called separately for each technology and then integrated by genotype refinement into a single phased reference panel. More details can be found in a previous study^39^.

The HCM independent target cohort was constructed by extracting non-EUR HCM samples (ICD-10: I42.1/I42.2) and a same number of randomly selected non-EUR controls matching age and sex from the UKBB genotype dataset. This resulted in a total of 152 samples.

The WGS data of the independent target cohort for AD were obtained from the ADNI database. A total of 808 whole genomes were downloaded from ADNI, for which we defined individuals with a diagnosis of ‘dementia’ as cases and ‘cognitively normal’ as controls.

### WGS data processing

The WGS data for HCM and COVID-19 were processed using the functional equivalence GATK variant-calling pipeline^148^, which was developed by the Broad Institute and plugged into our data and task management system Trellis. The human reference genome build was GRCh38. We used BWA-MEM (version 0.7.15) to align reads, Picard 2.15.0 to mark PCR duplicates and GATK 4.1.0.0 for base quality score recalibration and variant calling using the ‘haplotypeCaller’ function. We also used FASTQC (version 0.11.4), SAMtools ‘flagstat’ (version 0.1.19) and RTG Tools ‘vcfstats’ (version 3.7.1) to assess the qualities of the FASTQ, BAM and gVCF files, respectively. In addition, we used ‘verifybamID’ in GATK 4.1.0.0 to estimate DNA contamination rates for individual genomes and removed samples with 5% or more contaminated reads.

### QCs of genotype data

We performed stringent QCs for the genotype data following the PRS tutorial (https://choishingwan.github.io/PRS-Tutorial/). For the GWAS summary statistics data (also referred to as the discovery or base data), genetic variants with low MAF and imputation information score (INFO) were removed. We used thresholds suggested in corresponding original papers: MAF < 0.0001, 0.001 and 0.0001 and INFO < 0.4, 0.6 and 0.6 for T2D, HCM and AD, respectively. We also excluded duplicated and ambiguous variants to guarantee the accuracy of PRS calculation.

For the individual-level genotype data (also referred to as the target data), we carried out both variant-level and individual-level QCs. For WGS data, we performed pre-QCs: we removed samples with kinship > 0.03, sample call rate < 0.97 or mean sample coverage ≤ 18×; genomic positions resided in low-complexity regions or ENCODE-blacklisted regions were removed; we filtered out genotypes in individual samples that were detected with too low or too high read coverages (read depth < 5 or read depth > 1,500); we required all calls to have genotype quality ≥ 20 and, for nonreference calls, a sufficient portion (>0.9) of reads was required to cover the alternate alleles.

For all target cohorts, we removed variants with INFO < 0.8 (for UKBB-based cohort), missing call rate > 0.01, MAF < 0.01 or Hardy–Weinberg equilibrium < 1 × 10^−6^. For variants with mismatching alleles between discovery and target data, we strand-flipped these alleles to their complementary ones. We further excluded individuals with genotyping rate < 0.01 or with extreme heterozygosity rate (that is, beyond 3 s.d. from the mean). Individuals with an up-to-second-degree relative (*π* > 0.125) within the cohort were also removed to prevent bias in prediction evaluation. Lastly, there were 2,176 (*n* = 1,088 cases, *n* = 1,088 controls), 134 (*n* = 81 cases, *n* = 53 controls), 1,839 (*n* = 919 cases, *n* = 920 controls) and 581 (*n* = 120 cases, *n* = 461) individuals passing the above QCs for T2D, HCM, AD and severe COVID-19 cohorts, respectively.

All independent target cohorts were processed and QCed using the same pipeline. After sample-level QCs, the final cohorts consisted of 2,749 samples (1,398 cases and 1,351 controls) for GoT2D, 62 samples (23 cases and 39 controls) for non-EUR UKBB and 469 samples (251 cases and 218 controls) for ADNI.

### PC analysis for genotype data

To characterize the population structure of target cohorts, PC analysis was performed after pruning (window size = 200 variants, sliding step size = 50 variants, LD *r*^2^ threshold = 0.25). The first ten PCs were retained as covariates in the downstream analysis.

### PLINK C+T PRS calculation

The cell-level C+T PRS was computed using PLINK, which is given by

$${\rm{PRS}}_{j}=\frac{{\sum }_{i\in {\rm{cCRE}}_{j}}{\beta }_{i}\times {G}_{i}}{P\times M},$$

where ${\rm{cCRE}}_{j}$ denotes cCREs within cell *j*, ${\beta }_{i}$ is the effect size of variant *i*, ${G}_{i}$ represents the number of effect alleles, *P* is the ploidy of the sample (2 for human) and *M* is the number of nonmissing variants. In the clumping phase, all index variants were forced to be drawn from the variants located within scATAC-seq peaks of individual cells using the ‘--clump-index-first’ option. Variants within 250 kb of the index variant and three LD thresholds (*r*^2^ = 0.1, 0.3 and 0.5) were considered for clumping. After constructing the index variant set, we applied multiple *P*-value thresholds (*P* = 1 × 10^−5^, 1 × 10^−4^, 1 × 10^−3^, 0.01, 0.05, 0.1 and 0.5) to compute PRSs, resulting in 21 PRSs calculated for each cell and each individual. We used the 1,000 Genomes Project samples to estimate the LD (out-sample estimation) for the simulation, HCM and severe COVID-19 cohorts because of their limited sample sizes, while using the target data (in-sample estimation) for other cohorts.

The standard C+T PRS was calculated using the same set of parameters as that used in computing cell-level PRS, except that all variants were considered without conditioning. The *P*-value and LD *r*^2^ thresholds were regarded as hyperparameters to be optimized in model selection.

### Model details of scPRS

The cell-level PRS matrix ${X}_{n}\in {{\mathfrak{R}}}^{M\times 21}(n\in 1,\ldots ,N)$ presents single-cell-resolved genetic risk features for each individual and it is input into the scPRS model to predict the disease risk. Here, *N* and *M* denote the numbers of individuals and cells, respectively.

scPRS consists of three modules (Fig. 1): the feature-embedding module, the graph convolutional network module and the readout module. The feature-embedding module takes normalized cell-level PRS ${X}_{n}$ as the input and uses a one-layer perceptron to reweight and integrate 21 PRS features per cell:

$${h}_{n}^{(0)}={X}_{n}\bullet {\rm{abs}}({W}_{0}),$$

where *W*_0_ denotes learnable model parameters, abs represents the absolute function and ${h}_{n}^{(0)}\in {{\mathfrak{R}}}^{M}$ represents the integrated features of *M* cells for individual *n*. According to the definition of PRS, larger values in *X*_*n*_ indicate higher disease risk. To maintain this interpretability throughout the modeling, we adopt the absolute function abs to enforce nonnegativity for *W*_0_.

We next seek to integrate PRS features across different cells to generate a final risk score. With the consideration of the dropout event and sparsity of scATAC-seq data and assuming that cells with similar low-dimensional embeddings should have comparable epigenomes and then similar genetic signals, we use a GNN^149^ to smooth and denoise single-cell-level PRS features. More specifically, on the basis of the pre-computed M-kNN graph *G*, the GNN module is defined as

$${g}_{v}^{(t+1)}=\frac{1}{{\mathrm{deg}} (v)}\sum _{u{{\in }}{\mathscr{N}}{(}v)}\left({\rm{abs}}\left({w}_{1}^{(t)}\right){h}_{u}^{(t)}+{\rm{abs}}\left({w}_{2}^{(t)}\right){h}_{v}^{(t)}\right),$$

$${h}_{v}^{(t+1)}={\rm{leaky}}\;{\rm{ReLU}}\left({g}_{v}^{(t+1)}\right),$$

where ${h}_{v}^{(t)}$ denotes the hidden feature of cell *v* at layer *t*, ${w}_{1}^{(t)}$ and ${w}_{2}^{(t)}$ are learnable parameters of layer *t*, deg denotes the degree of each node or cell and ${\mathscr{N}}{(}v)$ represents the neighbors of cell *v* in the M-kNN graph *G*. The leaky ReLU activation function is defined as

$${\rm{Leaky}}\;{\rm{ReLU}}(x)=\max (\alpha \times x,x),$$

where $\alpha =0.1$ is used in this study. Note that the absolute function is also adopted to induce nonnegativity to model weights.

Lastly, we design a readout module to map GNN-smoothed hidden features to the phenotype leveraging a one-layer perceptron:

$$y=\sigma \left(\beta \bullet {h}^{(T)}+b\right),$$

where $\beta \in {{\mathfrak{R}}}^{M}$ represents the learnable regression coefficients indicating cell importance to prediction, *T* is the number of total layers in GNN, *b* is the bias term and $\sigma$ is the sigmoid function for binary classification and the identify function for regression.

### Optimization of scPRS

To train scPRS for disease prediction, we adopt the binary cross-entropy (BCE) loss and additional regularization functions for enhancing predictive power and model interpretability. The loss function ${\mathcal{L}}$ of scPRS is defined as

$${\mathcal{L}}{{=}}\frac{1}{N}\sum _{n}\left({y}_{n}\log ({p}_{n})+(1-{y}_{n})\log (1-{p}_{n})\right)+{\lambda }_{1}{{||}\beta {||}}_{1}+{\lambda }_{2}{{||}\beta {||}}_{2}+{\lambda }_{3}{\beta }^{T}{G}_{L}\beta,$$

where ${y}_{n}\in \{\mathrm{0,1}\}$ is the true disease label for individual *n*, ${p}_{n}\in [\mathrm{0,1}]$ is the scPRS-predicted disease probability and ${{||}\bullet {||}}_{1}$ and ${{||}\bullet {||}}_{2}$ represent *L*_1_ and *L*_2_ norms, respectively. We also add a Laplacian regularization term based on the symmetric normalized Laplacian matrix *G*_*L*_, which is defined as

$${G}_{L}={D}^{\frac{1}{2}}(D-A){D}^{-\frac{1}{2}},$$

where *D* and *A* denote the degree and adjacency matrices of the cell–cell similarity graph *G*, respectively. We use hyperparameters ${\lambda }_{1}$, ${\lambda }_{2}$ and ${\lambda }_{3}$ to balance across different regularization terms.

scPRS was trained by minimizing the loss ${\mathscr{L}}$ using the Adam algorithm^150^ with a learning rate of 1 × 10^−3^ and batch size of 32. We trained scPRS for 200 epochs. Multiple sets of hyperparameters were considered in model selection, including $T\in \{\mathrm{0,1,2}\}$, ${\lambda }_{1}\in \{\mathrm{0,1,10}\}$, ${\lambda }_{2}\in \{\mathrm{1,10,50,100,250,500,750}\}$, ${\lambda }_{3}\in \{\mathrm{0.01,0.1,0.5,1,2.5,5,10,50,100}\}$ and M-kNN neighbor number $k\in \{\mathrm{25,50}\}$. We also selected between CCA-based and Harmony-based cell–cell similarity networks for T2D and AD.

In prediction evaluation, we randomly partitioned the dataset into training, validation and testing sets comprising 60%, 20% and 20% of samples, respectively. We trained different scPRS models with all possible combinations of hyperparameters and assessed their performance (measured by AUROC) on the validation dataset. We selected the model yielding the best performance on the validation set and reported its performance on the held-out test set. This process was repeated ten times with different random seeds to assess the robustness of the model. Predictive performance was evaluated using both the AUROC and the AUPRC.

In cell prioritization, we conducted fivefold cross-validation, which was repeated five times. The best hyperparameter set was then selected on the basis of the average AUROC score. The final model was trained with this optimal hyperparameter set on the entire dataset. To examine the variability of cell weights learned from model training, we trained 100 models using different random seeds.

For the regression task, the mean squared error was used as the loss function instead of BCE. The model performance was evaluated based on the Pearson correlation between true and predicted values.

### Calculation of nonpeak and peak PRS

Similar to the cell-level PRS, the calculation of nonpeak PRS was based on PLINK C+T, using only variants outside of scATAC-seq peaks as the index variants. A total of 21 nonpeak PRSs were computed and integrated in scPRS+, corresponding to different combinations of C+T parameters: *P* ∈ {1 × 10^−5^, 1 × 10^−4^, 1 × 10^−3^, 0.01, 0.05, 0.1, 0.5} and *r*^2^ ∈ {0.1, 0.3, 0.5}. For scPRS+ (integrating cell-level PRSs and nonpeak PRSs) and scPRS+covar (integrating cell-level PRSs, nonpeak PRSs, age, sex and ten PCs), we concatenated additional features to latent cell features ${h}^{(T)}$ at the final GNN layer.

In calculating the single-cell-type peak PRS, only variants located within cell-type peaks were used to select the index variants, where the same 21 combinations of C+T parameters were adopted. A multi-cell-type PRS was further built by combining all single-cell-type PRSs (*n* = 21 × *n*_cell type_) using LR. LR was trained on the training data and the performance was reported on the testing data.

### Implementation details on LDpred2, Lassosum and PolyPred

We implemented LDpred2 and Lassosum following the bigsnpr tutorial (https://privefl.github.io/bigsnpr/articles/LDpred2.html). Three LDpred2 models were implemented” the infinitesimal model (LDpred2-inf), grid model (LDpred-inf) and auto model (LDpred2-auto). All model hyperparameters were selected on the basis of recommendations provided in the tutorial. To ensure a fair comparison, we maintained the same dataset splits (that is, training, validation and test sets) as those used in scPRS. For PLINK C+T, LDpred2-grid and Lassosum, the best model hyperparameters were determined on the basis of predictive performance on the validation dataset.

For a fair comparison, we used scATAC-seq peaks as the functional annotation for variants in PolyPred and adopted the same GWASs as those used in scPRS to compute prior causal probabilities^38^. We implemented PolyPhred following the manual provided by the authors (https://github.com/omerwe/polyfun/wiki).

Unlike C+T, more advanced PRS methods, including LDpred2, Lassosum and PolyPred, inherently optimize *r*^2^ and *P*-value cutoffs to select an optimal set of variants for PRS computation. This flexibility in optimization is a key innovation of these approaches.

### Benchmark on independent target cohorts

Because the original GWAS discovery cohorts for T2D and AD overlapped with GoT2D and ADNI, respectively, to prevent information leakage, we adopted the UKBB GWAS^151^ as new summary statistics for T2D and AD, which were independent from the new target cohorts. We then trained new scPRS models on the basis of original target cohorts. For C+T, LDpred2-grid and Lassosum, model hyperparameters were optimized on the basis of original target cohorts. For scPRS, hyperparameters were selected using fivefold cross-validation of the original target cohorts. All PRS approaches were tested on the basis of new independent target cohorts.

### Prioritization of disease-relevant cells and cell types using scPRS

The mapping from input PRS features *X* to latent cell features ${h}^{(T)}$ monotonically increases as a result of the design principle of scPRS, where weights in the embedding and GNN modules are constrained to be nonnegative. This features facilitates model interpretation: a larger value of ${\beta }_{m}$ denotes a higher enrichment of genetic risk within that cell, thereby informing disease–cell relevance. To account for the variability of learned cell weights, we trained 100 scPRS models and compared the distribution of ${\beta }_{m}$ for individual cells with that of top-ranking weights (that is, the top 15% of all cell weights per repeat) using a one-sided *t*-test. This comparison was conducted for each cell in the dataset. We defined disease-relevant cells as those cells whose adjusted *P* values (using the Benjamini–Yekutieli procedure) were less than 0.1. Roughly speaking, scPRS prioritizes cells whose weights are consistently larger than those of the majority of cells.

To get more biological insights, we examined the enrichment of scPRS-prioritized cells within each cell type using a Fisher’s exact test. The disease-relevant cell types were defined as those cell types whose adjusted *P* values (using the BH procedure) were less than 0.1.

### Simulation details

Using the PBMC multiome data downloaded from 10x Genomics, we first conducted the differential accessibility analysis to identify monocyte-specific scATAC-seq peaks. In this study, we defined monocytes as the total set of CD14/CD16 monocytes and dendritic cells considering their shared heritability^152^. We identified differentially accessible regions (DARs) within monocytes using the top 1,500 marker peaks per cell subtype. Next, leveraging a monocyte count GWAS^22^, we computed PLINK C+T PRS conditioned on the variants located within monocyte DARs for a WGS cohort^23^ (*n* = 401). Raw C+T PRS outputs were further standardized to mean = 0 and variance = 1, yielding the ‘ground truth’ of monocyte count for this cohort.

To introduce randomness, we added a noise term to the simulated monocyte count:

$$\widetilde{y}=y+\varepsilon,$$

where $\varepsilon \sim {\mathscr{N}}\left(0,{\sigma }^{2}\right)$. In this study, we used $\sigma \in \{\mathrm{0,0.25,0.5,1,3,5,7}\}$. We trained scPRS on the basis of these simulation datasets with and without noises to evaluate its capacity in identifying phenotype-associated cells.

### SCAVENGE

We used SCAVENGE^46^ as a benchmark for prioritizing disease-relevant cells. Following the SCAVENGE tutorial (https://sankaranlab.github.io/SCAVENGE/articles/SCAVENGE), we calculated trait relevance scores (TRSs) for individual cells, indicative of their association with the disease. Cells were prioritized by SCAVENGE if their TRSs were above 95% of all TRSs. As in the scPRS analysis, we evaluated the enrichment of selected cells within each cell type using the Fisher’s exact test.

### Stratified LDSC

Partitioned heritability analysis was carried out using sLDSC as previously described^45^. Heritability was quantified within the total set of snATAC-seq peaks identified for each of the left-ventricle cell types. Genetic enrichment for a particular cell type was defined by calculating the captured heritability per unit of sequence within the total set of identified snATAC-seq peaks for that cell type, compared to the genome overall. *P* values were calculated as previously described^45^; nominal significance (*P* < 0.05) was taken to be indicative of true enrichment.

We conducted sLDSC using the same GWAS and scATAC-seq datasets as those used in scPRS for HCM and severe COVID-19, for which no existing sLDSC results were available. For AD, the original sLDSC^35^ was performed on the same GWAS and scATAC-seq dataset. For T2D, the original sLDSC^44^ was carried out on the same scATAC-seq dataset but used a larger GWAS^153^. We chose to report the results of sLDSC applied to discovery GWAS to optimize its power, given the larger sample size of discovery GWAS compared to target cohort.

### Identification of disease-relevant cCREs

As the first step of the layered multiomic analysis (Fig. 5a), we identified differentially accessible cCREs within each scPRS-prioritized cell type using Signac^154^. Specifically, we used the FindMarker function to compare peaks within scPRS-prioritized cells (per cell type) against all unselected cells in the dataset as background, with parameters test.use = ‘LR’, latent.vars = ’peak_region_fragments’, min.pct = 0.02,and logfc.threshold = 0.1. Significant peaks (adjusted *P* < 0.1 based on BH correction) with a positive log_2_ FC were defined as differentially accessible cCREs. Next, leveraging the discovery GWAS summary statistics, we conducted MAGMA^74^ analysis for these differentially accessible cCREs per cell type, with gene-model = ‘multi’. MAGMA is a widely used tool for gene-level and region-level genetic association analysis based on GWAS summary data. It is designed to test genetic associations of predefined genes or regions with diseases or traits by aggregating variant-level GWAS statistics while accounting for LD. We defined disease-relevant cCREs (T2D-cCREs and AD-cCREs) as those cCREs with adjusted MAGMA *P* < 0.1 based on BH correction. We expanded our analysis to involve all nominally significant cCREs (MAGMA *P* < 0.05) for HCM, as no cCRE passed the multiple-testing correction.

### Mapping cCRE–gene links

We mapped cCREs to their target genes on the basis of two complementary strategies. First, we adopted the closest-gene strategy^155^ and assigned each cCRE to its closest gene. In addition, we added more distant genes on the basis of a coaccessibility analysis using Cicero^75^ and linked each cCRE to those genes whose TSS peak displayed coaccessibility with the cCRE above 80% of all interactions. For each scPRS-prioritized cell type, the expressed genes mapped to disease-relevant cCREs within that cell type defined the repertoire of disease candidate genes.

### Enrichment of disease-associated variants within scPRS-cell-specific peaks

Per disease-relevant cell type, we performed clumping within differentially accessible peaks in scPRS-prioritized cells to remove redundant variants. Multiple LD *r*^2^ thresholds (*r*^2^ = 0.1, 0.3 and 0.5) were tested. Leveraging the clumped variant set, we examined the enrichment of disease-associated variants (GWAS *P* < 5 × 10^−8^) within scPRS-cell-specific peaks by comparing it to the genome-wide distribution.

### TF-binding motif analysis

The TF-binding motif analysis was performed using GimmeMotifs^156^. The differential motifs between disease-relevant cCREs and all peaks within the corresponding cell type were identified using the ‘gimme motif’ command with options f = 0.5 and s = 0. AUROC was adopted to quantify the motif enrichment.

### Network analysis

We downloaded the human PPIs from STRING (version 12.0)^98^, comprising 19,622 proteins and 6,857,702 interactions. High-confidence PPIs (combined score > 700) were extracted for downstream analysis, including 16,185 proteins and 236,000 interactions. To mitigate bias from hub proteins^157^, we applied the random walk with restart algorithm with a restart probability of 0.5. This produced a smoothed network after retaining the top 5% predicted edges (*n* = 6,243,766). Next, we used the Louvain method^158^ to decompose the network into different modules. Following algorithm convergence, we obtained 1,261 modules with an average size of 13 nodes.

The enrichment of genes of interest within each module was tested using the hypergeometric test. Modules with adjusted *P* < 0.1 based on BH correction were considered significant.

### Sequence deep learning model design and training

The sequence-based deep learning model was trained to predict ATAC-seq peaks across various cell types on the basis of the DNA sequence. Specifically, the sequence model takes a 2,000-bp DNA sequence as the input and outputs the peak status of the centered 200 bp for different cell types. The peak label for a specific cell type is 1 if over 50% of the centered 200 bp is overlapped by an ATAC-seq peak within that cell type and 0 otherwise. Model structure follows the Beluga architecture^78^, except its outputs correspond to different cell types within the tissue of interest.

ATAC-seq peaks within chromosomes 6 and 7 and chromosomes 8 and 9 were held out as validation and test data, respectively. Peaks in other chromosomes were used as training data. Genomic regions annotated by the ENCODE blacklist^159^ were excluded from analysis. We adopted the BCE loss as the objective function. The sequence model was trained using the stochastic gradient descent algorithm with a weight decay coefficient of 1 × 10^−6^, momentum of 0.9, learning rate of 0.08 and batch size of 64. The model was implemented using Selene^79^, a PyTorch-based library for sequence deep learning modeling. In this study, we trained separate sequence models using different scATAC-seq datasets.

### Prediction of variant effects using sequence deep learning model

We used the sequence model to predict the impact of genetic variants on cCREs across diverse cell types. For a given cell type *c* and variant *v* (from reference allele to alternative allele), the model predicts the status of cCRE *y*_ref,*c*_ and *y*_alt,*c*_ for sequences centered on the reference and alternative alleles, respectively. We define the functional effect of variant *v* in cell type *c* as *y*_*v*,*c*_ = *y*_alt,*c*_ − *y*_ref,*c*_, representing how the variant alters cCRE in this cell type. To achieve a global evaluation of functional scores, we introduce the *Z*_*v*,*c*_ score, which normalizes *y*_*v*,*c*_ as *Z*_*v*,*c*_ = (*y*_*v*,*c*_ − *μ*)/*σ*, where *μ* and *σ* denote the mean and s.d. of all variant scores, respectively. The *Q*_*v*,*c*_ score is further defined as the quantile of |*Z*_*v*,*c*_| among all variants. A higher *Q* score indicates a larger functional effect within a specific cell type.

### Benchmarking sequence model prediction

To benchmark the sequence model prediction on variant effects against QTL analysis (eQTL or caQTL), we compared the absolute *Z* scores computed by the sequence model between QTLs and non-QTLs using a two-sided *t*-test. The *t* statistics was used to measure the enrichment of functional variants defined by the sequence model within QTLs.

As the second benchmarking, we used SNP2TFBS^89^ to predict the effects of variants on altering TFBS affinity. The binding affinities for different TFs were averaged for each studied variant to estimate its overall effect. Given a particular quantile cutoff, variants were split into two groups according to their *Q* scores. We then compared the averaged SNP2TFBS scores between these two groups of variants using a two-sided *t*-test. We report the *t* statistic, which indicates the enrichment of TFBS-disrupting variants within sequence-model-defined functional variants.

### Variant effect within disease-relevant cCREs

We compared the abundance of functional disease-associated variants (GWAS *P* < 0.05) within disease-relevant cCREs against the background using a Fisher’s exact test. Similarly, the functional variants were defined as those with *Q* scores above a given cutoff (multiple cutoffs applied). The odds ratio (OR) was adopted to measure the enrichment of functional variants within disease cCREs.

### Fine-mapping disease risk variants

We used three approaches to fine-map disease risk variants: the sequence deep learning model, QTL and TFBS. A 0.8 quantile cutoff was adopted to define functional variants on the basis of the sequence model in fine-mapping. In addition to SNP2TFBS, motifbreakR^91^ was used to predict variant disruption on TF binding. A positive averaged SNP2TFBS score or a strong-effect motifbreakR score was used to define a disrupting variant. We excluded missense and loss-of-function variants and variants with GWAS *P* ≥ 0.05 from fine-mapping.

### iPS cell reprogramming

iPS cells were reprogrammed from PBMCs using Sendai virus (CytoTune iPS 2.0 Sendai Reprogramming Kit) as previously described^160^. Three clones were generated per subject, karyotyped (KaryoStat, Thermo Fisher Scientific), determined to be free of *Mycoplasma* and evaluated by immunohistochemistry for expression of pluripotency markers TRA-1-60 (LifeTech, MA1023) and SSEA4 (LifeTech, MA1021). Cells were maintained under feed-free conditions in mTeSR (StemCell Technologies, 5850) or Essential 8 medium (Fisher, A1517001) and stored in liquid nitrogen.

### CDM differentiation and drug treatment

As previously described^161^, iPS cells were plated on Matrigel and cultured in StemMACS iPS-Brew XF (MACS Miltenyi Biotec, 130-104-368) until the final passage in Essential 8 medium (Fisher, A1517001). CDM differentiation was induced at 60–80% confluency, with culture in RPMI medium (Gibco/LifeTech, 11875-119) plus B27 supplement lacking insulin (Gibco/LifeTech, A1895601). Then, 6 µM CHIR-99021 (Fisher, NC0976209) was added on day 0 and 6 µM IWR1 (Fisher, NC1319406) was added on day 3. Beginning on day 7, the medium was changed every other day using RPMI medium supplemented with B27 containing insulin (Gibco/LifeTech 17504-044). Upon commencement of beating (around day 15), cells underwent purification by a 3-day glucose starvation (RPMI medium without glucose (Gibco/LifeTech, 11879-020) supplemented with insulin-containing B27), a 1-day recovery in glucose-containing medium and subsequent replating (dissociated in TrypLE, Fisher, 50-591-353). Cells were then maintained in RPMI medium supplemented with insulin-containing B27 until approximately day 30. After differentiation, drug treatment occurred at 0 and 24 h and samples were assayed at 48 h. Cells were treated with 250 nM MYK-461 (Cayman Chemical, 19216-5mg), 400 nM or 1 μM omecamtiv mecarbil (Selleckchem, Fisher, NC1069600) or DMSO.

### RNA-seq library preparation, sequencing, QC and expression matrix generation

RNA was extracted from iPS cells or CDMs (RNeasy, Qiagen). Illumina RNA-seq libraries (TruSeq Stranded Total RNA LP Gold) were prepared on the Bravo (Agilent), pooled and sequenced (NovaSeq 6000, paired-end, 100 bp)^27^. Where possible, drug treatment conditions for the same differentiation were kept together in batches, while replicate differentiations for the same iPS cell lines were split apart and HCM and control samples were distributed across batches. Reads were aligned to hg38 (STAR). PC analysis on CDM and iPS samples separately returned no outlier samples (defined as *Z* score of PC1 > 3). Library QC was assessed using fastp, fastQC, STAR and Picard metrics. Samples were flagged for poor QC by the following metrics: G+C content after filtering outside of 20–80% (fastp), duplication rate greater than 40% (fastp), uniquely mapped read pairs (fragments) < 20 million (STAR), mean reads (average of forward and reverse) < 20 million (fastQC), ribosomal RNA bases > 20% (Picard), coding plus UTR (untranslated region) < 50% (Picard) and uniquely mapping fragments < 60% (STAR). Samples with more than one flag were removed. CDM and iPS cell samples were subsequently processed separately. Reads were computed as counts per million (edgeR), corrected for library preparation batch (combat-seq) and normalized by the trimmed mean of *M* values (TMM; edgeR) to generate the final expression matrix. For samples with biological replicates, TMM counts were averaged. PC analysis was performed and PC1 was assessed for Spearman correlation with the following metadata: percent G+C content (fastp), mean reads (average of forward and reverse) in millions (fastQC), percent ribosomal RNA bases (Picard), uniquely mapped fragments in millions (STAR), duplication rate (fastp), percent coding or UTR (Picard), library preparation batch and sequencing pool. The maximum absolute value for spearman correlation between PC1 and the library metadata was 0.08 for CDM samples, indicating good QC with technical artifacts having minimal influence on the dataset. iPS cell samples had higher correlation for three metrics (0.26 with G+C content, 0.22 with duplication rate and 0.11 with percent coding or UTR), with the remaining having less than an absolute value of 0.04.

### Differential expression analysis

Raw data were input into DESeq2 (ref. ^162^) as required to compare gene expression between HCM cases and controls across different conditions. Gene counts were averaged across replicates. Sample sex and ancestry were included as covariates in the analysis.

### Allelic imbalance analysis in rs7922621 prime-edited microglia

The rs7922621 prime-edited WTC11 clones were obtained from our previous study^108^ and microglia were differentiated accordingly. Total RNA was isolated from wild-type and prime-edited microglia using the RNeasy plus mini kit (Qiagen, 74034). Briefly, 400 ng of total RNA was reverse-transcribed using the iScript complementary DNA (cDNA) synthesis kit (Bio-Rad, 1708891). The cDNA region containing phased heterozygous SNP of *ANXA11* (rs2573353 in WTC11)^163^ was amplified using the following primers: WTC-ANX-F, AGGTCCAATAATCCCTGCTGA; WTC-ANX-R, CCATGGTGCTCGGCTAATTT. The PCR products were purified by agarose gel extraction, followed by the addition of Illumina adaptors and deep sequencing. Reads were aligned to the sequence of either allele and counted if the 100-bp regions surrounding rs2573353 were exactly matched.

### Differentiation of TMEM119–Tdtomato reporter cell line iMGs

iPS cells stably expressing a TMEM119–tdTomato reporter transgene were first differentiated into fibroblast-like cells using a previously established method^110,111^. TMEM119–tdTomato fibroblasts were seeded onto 96-well plates (Corning) coated with 0.1% gelatin and Matrigel in fibroblast medium (DMEM with 10% FBS and 1% penicillin–streptomycin). After 48 h, the cells were transduced with 200 μl of two different concentrated retroviruses to overexpress the human *PU.1* and *CEBPA* per 96-well well with 5 μg ml^−1^ polybrene in fibroblast medium. Then, 24 h after transduction, the medium was switched to DMEM with 5% FBS, 10 ng ml^−1^ human macrophage colony-stimulating factor (M-CSF) and 10 ng ml^−1^ interleukin 34 (IL-34) and refreshed every 3 days thereafter. iMGs expressing the TMEM119–tdTomato reporter were used for experiments 14 days after viral transduction.

### siRNA transfection

siRNAs (Thermo Fisher) at a concentration of 30 nM were transfected into iMGs on day 14 using Lipofectamine RNAiMAX transfection reagent (Thermo Fisher Scientific, 13778075) in complete iMG medium (DMEM + 5% FBS, 10 ng ml^−1^ M-CSF and 10 ng ml^−1^ IL-34). After 24 h, the medium was refreshed with complete iMG medium; after an additional 24 h (48 h after transfection), cell cultures were collected for RT–qPCR or pHrodo analysis.

### pHrodo phagocytosis assay

iMGs cultured in 96-well plates (Corning) coated with Matrigel and gelatin were incubated with 10 μg of pHrodo green *Escherichia coli* bioparticles (Inucyte) for 15 min at 37 °C. Wells were then washed with PBS and were longitudinally imaged with Molecular Devices ImageExpress at 30-min intervals for the initial 2 h and 1-h intervals thereafter up to 24 h after the start. The 2-h time point was selected for downstream analysis. ImageJ software was used for quantification of individual replicates across conditions on the basis of the colocalization of TMEM119–Tdtomato and pHrodo green.

### Reporting summary

Further information on research design is available in the Nature Portfolio Reporting Summary linked to this article.

## Online content

Any methods, additional references, Nature Portfolio reporting summaries, source data, extended data, supplementary information, acknowledgements, peer review information; details of author contributions and competing interests; and statements of data and code availability are available at 10.1038/s41587-025-02725-6.

## Supplementary information

Supplementary Information Supplementary Figs. 1–11.

Reporting Summary

Supplementary Table 1 T2D-relevant variants, cCREs and genes identified in different cell types.

Supplementary Table 2 HCM-relevant variants, cCREs and genes identified in different cell types.

Supplementary Table 3 Differential expression analysis for HCM iPS cell-derived CDM RNA-seq data.

Supplementary Table 4 AD-relevant variants, cCREs and genes identified in different cell types.

Supplementary Table 5 GO analysis for AD candidate genes.

Supplementary Table 6 Summary of variant numbers for different diseases.

## Data Availability

The PBMC multiome dataset is available from 10x Genomics (https://support.10xgenomics.com/single-cell-multiome-atac-gex/datasets/1.0.0/pbmc_granulocyte_sorted_10k). The single-cell multiome data (snRNA-seq and snATAC-seq coassay) of the human left ventricle and lung are publicly accessible through ENCODE 4 (https://www.encodeproject.org/single-cell/?type=Experiment&assay_slims=Single+cell&status=released). All other scATAC-seq datasets were obtained from their original publications^33,35^. The WGS data used in simulation are available from a previous study^23^. Individual-level genotype–phenotype data for T2D and AD were sourced from the UKBB. The WGS and iPS cell RNA-seq data for HCM are available from a previous study^27^. The COVID-19 WGS and clinical data are available upon request from the corresponding authors (P.S.T. and M.P.S.); these data are not publicly available because of US Government and Department of VA restrictions relating to participant privacy and consent. The independent target cohorts for T2D, HCM and AD are accessible through the European Genome-Phenome Archive (EGAD00001002247), UKBB and ADNI (https://adni.loni.usc.edu/data-samples/adni-data/), respectively. The HCM snRNA-seq dataset was obtained from a previous study^101^. All GWAS summary statistics data were acquired from their original publications^22,29–31^. The GTEx and islet eQTL datasets were downloaded from the eQTL catalog (https://www.ebi.ac.uk/eqtl/). Other eQTL and caQTL datasets were obtained from their original publications^60,85,88,164^. The reference human genomes (hg19 and hg38) are available online (https://hgdownload.soe.ucsc.edu/downloads.html#human).

## Code availability

The source code and tutorial for scPRS are available from GitHub (https://github.com/szhang1112/scPRS).
