# Supplementary material for: Single-cell polygenic risk scores dissect cellular and molecular heterogeneity of complex human diseases
Source: Nat Biotechnol. 2025 Jul 25;44(5):845–61. doi: 10.1038/s41587-025-02725-6 (PMC13180658; doi:10.1038/s41587-025-02725-6)
Supplement: Supplementary file 1 — Supplementary Figs. 1–11. [file 41587_2025_2725_MOESM1_ESM.pdf]

nature biotechnology

Supplementary information

https://doi.org/10.1038/s41587-025-02725-6

# Single-cell polygenic risk scores dissect cellular and molecular heterogeneity of complex human diseases

In the format provided by the authors and unedited

## Supplementary Tables 1 – 6 (provided as separate Excel files)

**Supplementary Table 1**. T2D-relevant variants, cCREs, and genes identified in different cell types.

**Supplementary Table 2**. HCM-relevant variants, cCREs, and genes identified in different cell types.

**Supplementary Table 3**. Differential expression analysis for HCM iPSC-cardiomyocyte RNA-seq data.

**Supplementary Table 4**. AD-relevant variants, cCREs, and genes identified in different cell types.

**Supplementary Table 5**. Gene ontology analysis for AD candidate genes.

**Supplementary Table 6**. Summary of variant numbers for different diseases.

## Supplementary Figures 1 – 11

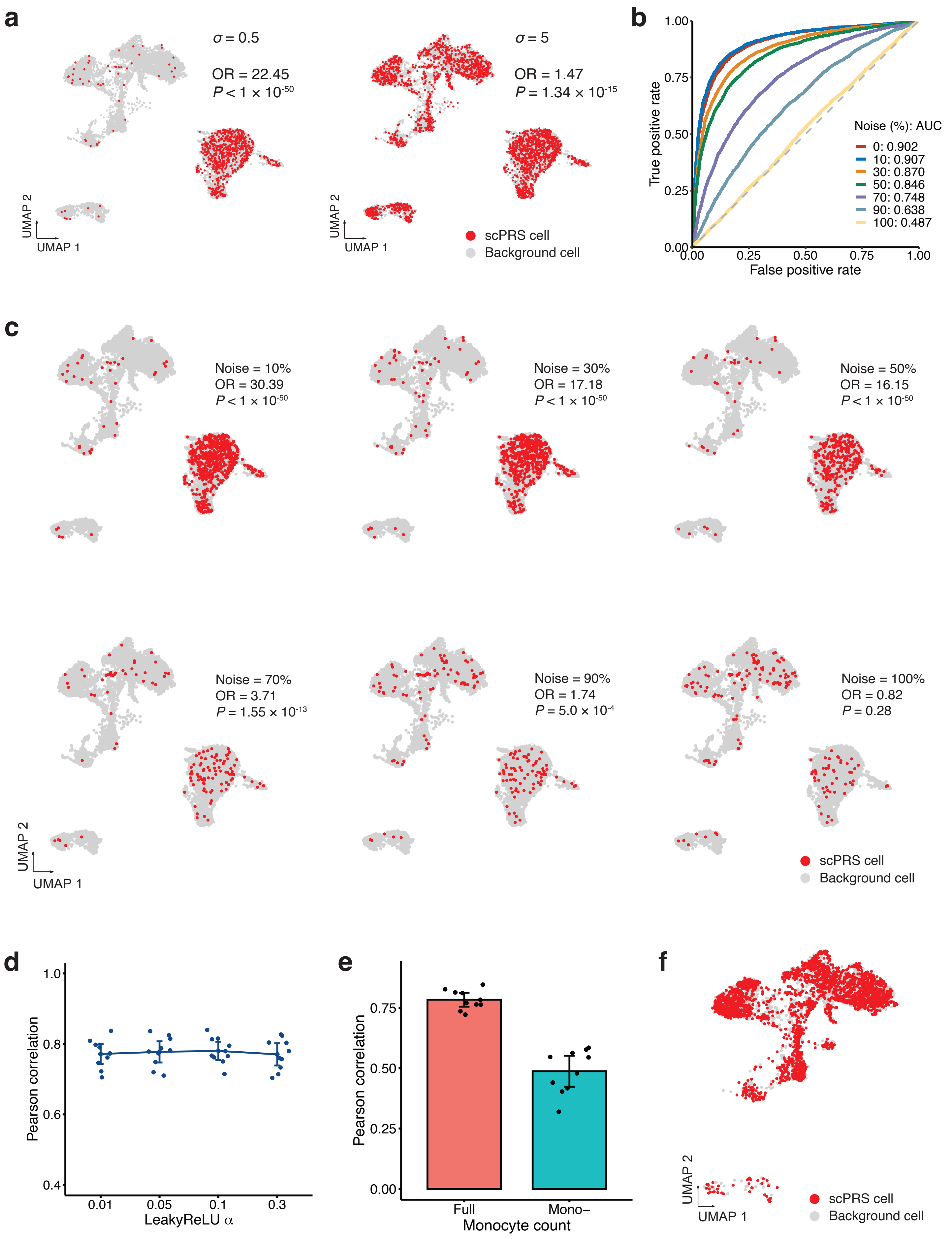
a
σ = 0.5
OR = 22.45
P < 1 × 10-50
σ = 5
OR = 1.47
P = 1.34 × 10-15
UMAP 2
UMAP 1
scPRS cell
Background cell
b
True positive rate
False positive rate
Noise (%): AUC
0: 0.902
10: 0.907
30: 0.870
50: 0.846
70: 0.748
90: 0.638
100: 0.487
c
Noise = 10%
OR = 30.39
P < 1 × 10-50
Noise = 30%
OR = 17.18
P < 1 × 10-50
Noise = 50%
OR = 16.15
P < 1 × 10-50
Noise = 70%
OR = 3.71
P = 1.55 × 10-13
Noise = 90%
OR = 1.74
P = 5.0 × 10-4
Noise = 100%
OR = 0.82
P = 0.28
d
Pearson correlation
LeakyReLU α
e
Pearson correlation
Full
Mono−
Monocyte count
f

**Supplementary Fig. 1 | Simulation results in different error and model settings. a**, Monocyte-count-relevant cells prioritized by scPRS (in red) in different noise settings. *P*-value and OR by two-sided Fisher's exact test. UMAP, uniform manifold approximation and projection; OR, odds ratio. **b,** The receiver operating characteristic (ROC) curves for cell prioritization with different peak noises. AUC, the area under the curve; %, percentage of peaks replaced by non-monocyte-specific peaks. **c,** Monocyte-count-relevant cells prioritized by scPRS (in red) in different peak noises. OR and *P*-value by two-sided Fisher's exact test. **d,** Pearson correlation between simulated and predicted monocyte counts with different LeakyReLU parameters. The training and testing procedure was conducted for 10 repeats with different random seeds. The mean and 95% confidence interval (CI) are denoted in the dot and error bar, respectively. $\alpha$ represents the negative slope of the LeakyReLU activation function used in scPRS. $\alpha$ = 0.1 is our initial setting. **e,** Predictive performance comparison between scPRS models trained using the full PBMC dataset and the dataset excluding monocytes. The training and testing procedure was conducted for 10 repeats with different random seeds. Mono-, the PBMC dataset excluding monocytes and cells containing >40 monocyte-specific peaks. The bar plot and error bar denote the mean and 95% CI, respectively. **f,** Monocyte-count-relevant cells prioritized by scPRS trained without monocytes.

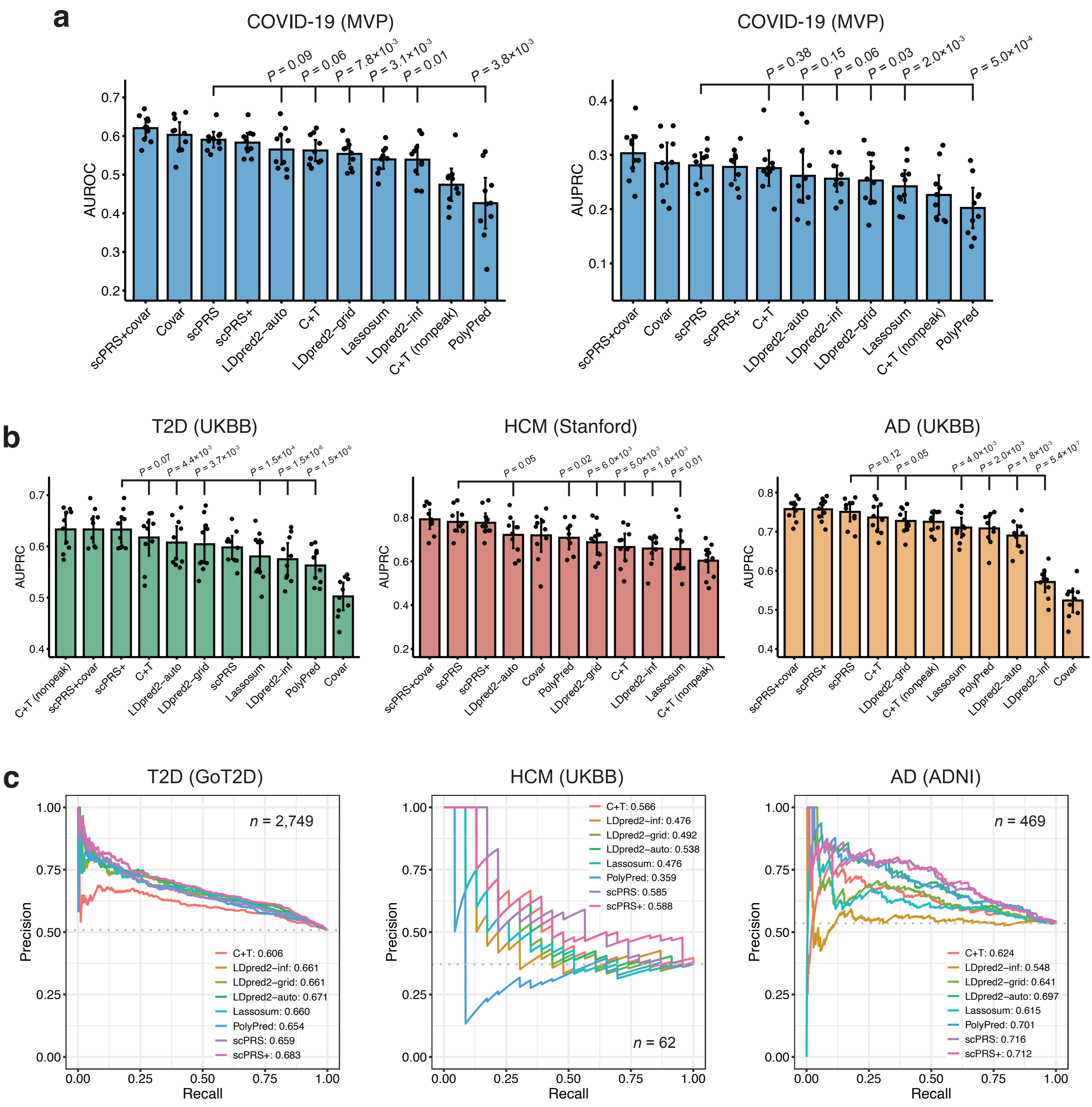


**Supplementary Fig. 2 | Benchmark results on disease prediction. a**, Predictive performance for severe COVID-19. The training and testing procedure was conducted for 10 repeats with different random seeds. Training, validation, and test dataset splits were kept identical across different methods to ensure a fair comparison. scPRS+, scPRS model integrating non-peak PRSs; scPRS+covar, scPRS model integrating non-peak PRSs and covariates; C+T, clumping and thresholding PRS; C+T (nonpeak), logistic regression model of non-peak C+T PRSs; Covar, logistic regression model of covariates. Performance comparison was conducted using one-sided paired *t*-test. The mean and 95% confidence interval (CI) are annotated using the barplot and error bar, respectively. AUROC, the area under the receiver operating characteristic curve; AUPRC, the area under the precision-recall curve. **b**, Barplots of AUPRC scores for T2D, HCM, and AD. The training and testing procedure was conducted for 10 repeats with different random seeds. Training, validation, and test dataset splits were kept identical across different methods to ensure a fair comparison. Performance comparison was conducted using one-sided paired *t*-test. The mean and 95% CI are annotated using the barplot and error bar, respectively. **c**, Precision-recall curves of different models evaluated on independent target cohorts. Performance of a random predictor is shown in the dashed gray line.

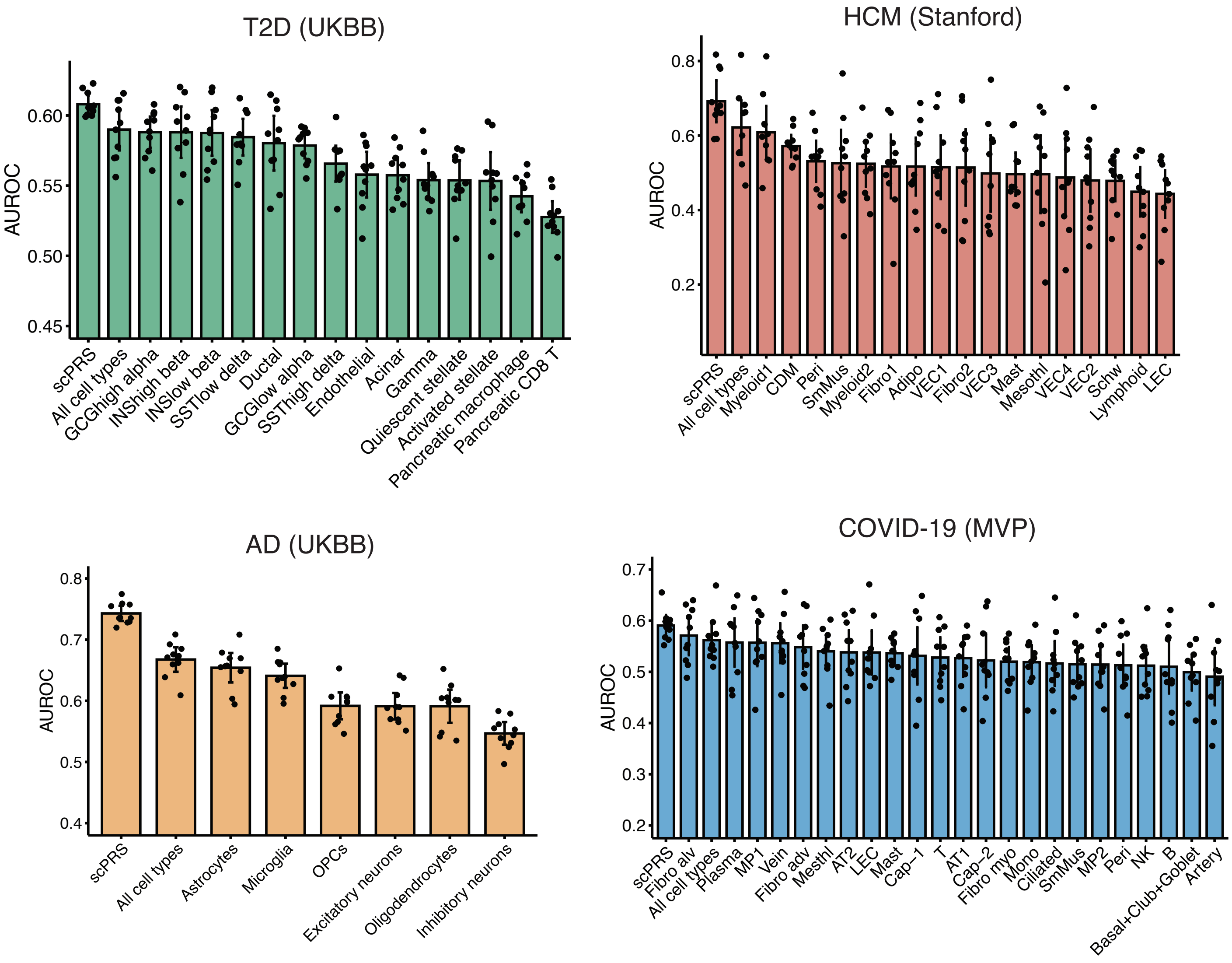


**Supplementary Fig. 3 | Predictive performance comparison between scPRS and cell-type-level PRSs.** The training and testing procedure was conducted for 10 repeats with different random seeds. The bar plot and error bar represent the mean and the 95% confidence interval (CI), respectively. AUROC, area under the receiver operating characteristic curve; CDM, cardiomyocyte; Fibro, fibroblast; LEC, lymphatic endothelial cell; Peri, pericyte; Schw, Schwann cell; SmMus, smooth muscle cell; VEC, vascular endothelial cell; Adipo, adipose; Mesothl, mesothelial cell; OPC, oligodendrocyte progenitor cell; Fibro alv, alveolar fibroblast; MP, macrophage; Fibro myo, myofibroblast; AT, alveolar type; Cap, capillary endothelial cell, mono, monocyte; NK, natural killer; Fibro adv, adventitial fibroblast.

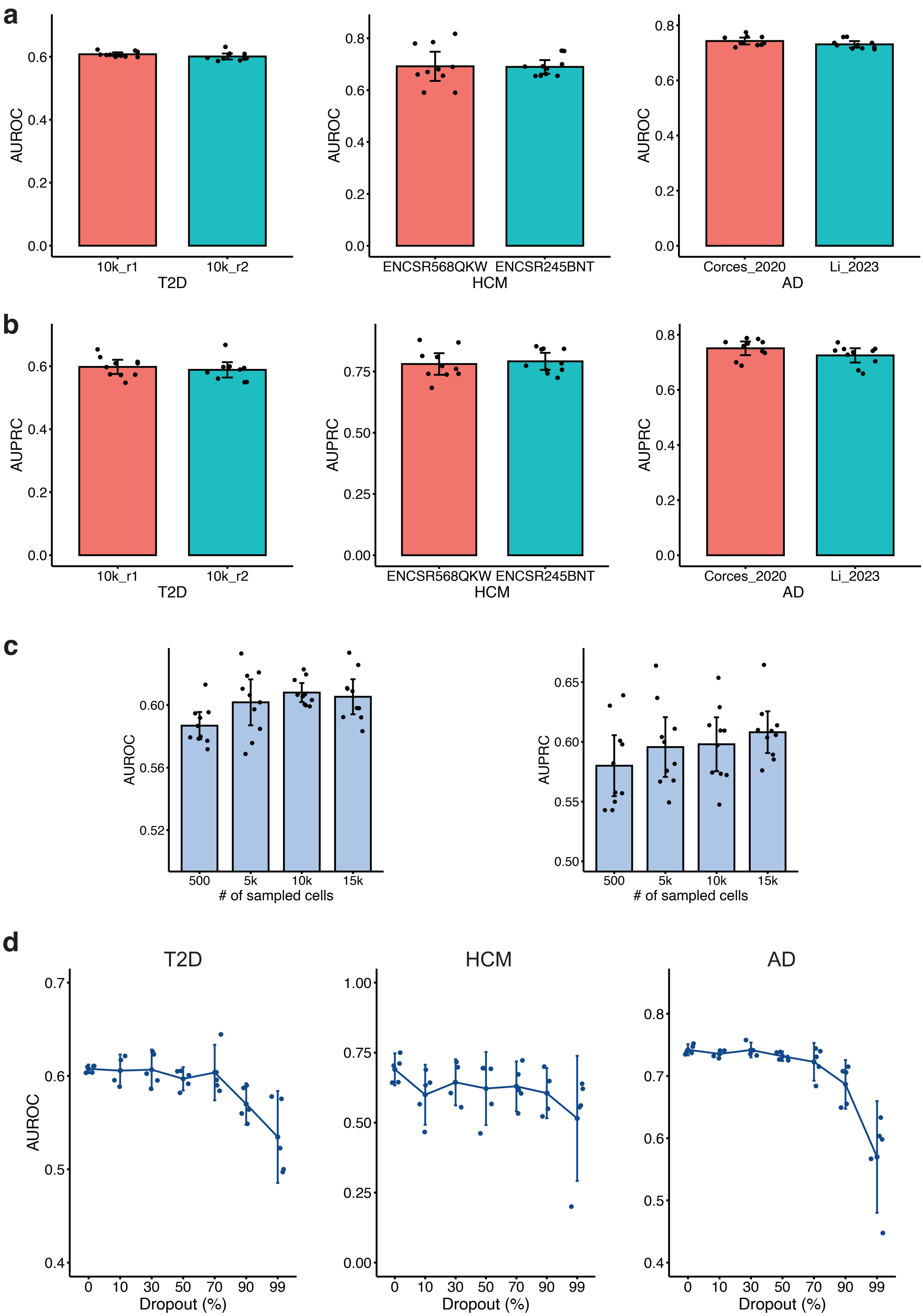
a
AUROC
10k_r1
10k_r2
T2D
ENCSR568QKW
ENCSR245BNT
HCM
Corces_2020
Li_2023
AD
b
AUPRC
10k_r1
10k_r2
T2D
ENCSR568QKW
ENCSR245BNT
HCM
Corces_2020
Li_2023
AD
c
AUROC
AUPRC
500
5k
10k
15k
# of sampled cells
d
T2D
HCM
AD
AUROC
Dropout (%)

**Supplementary Fig. 4 | Predictive performance evaluation in different settings. a-b,** Predictive performance of scPRS models using different reference scATAC-seq datasets, evaluated by AUROC (**a**) and AUPRC (**b**), respectively. The training and testing procedure was conducted for 10 repeats using different random seeds. AUROC, area under the receiver operating characteristic curve; AUPRC, area under the precision-recall curve; 10k_r1, the first repeat of randomly sampling 10,000 cells; 10k_r2, the second repeat of sandomly sampling 10,000 cells. ENCSR568QKW and ENCSR245BNT denote different donor identifiers. The bar plot and error bar represent the mean and 95% confidence interval (CI), respectively. **c,** Predictive performance of T2D scPRS models with different cell numbers. **d,** Predictive performance of scPRS models with different input PRS dropout rates. The training and testing procedure was conducted for five repeats with different random seeds. Only one PRS feature was left with a 99% dropout rate. The mean and 95% CI are denoted in the dot and error bar, respectively.

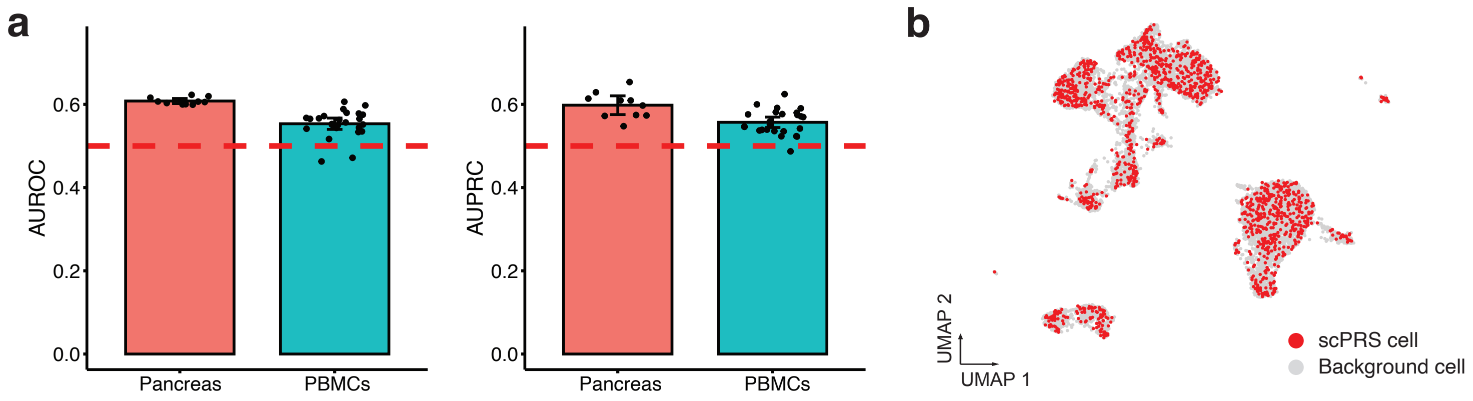


**Supplementary Fig. 5 | Negative control results on T2D scPRS trained based on the PBMC scATAC-seq dataset. a**, Predictive performance comparison between T2D scPRS models trained on the pancreas and the PBMC scATAC-seq datasets. The training and testing procedure was conducted for 10 repeats with different random seeds. The bar plot and error bar represent the mean and the 95% confidence interval (CI), respectively. The red dashed line denotes the score of 0.5. AUROC, area under the receiver operating characteristic curve; AUPRC, area under the precision-recall curve. **b**, Cells prioritized by T2D scPRS trained on the PBMC scATAC-seq data. UMAP, uniform manifold approximation and projection.

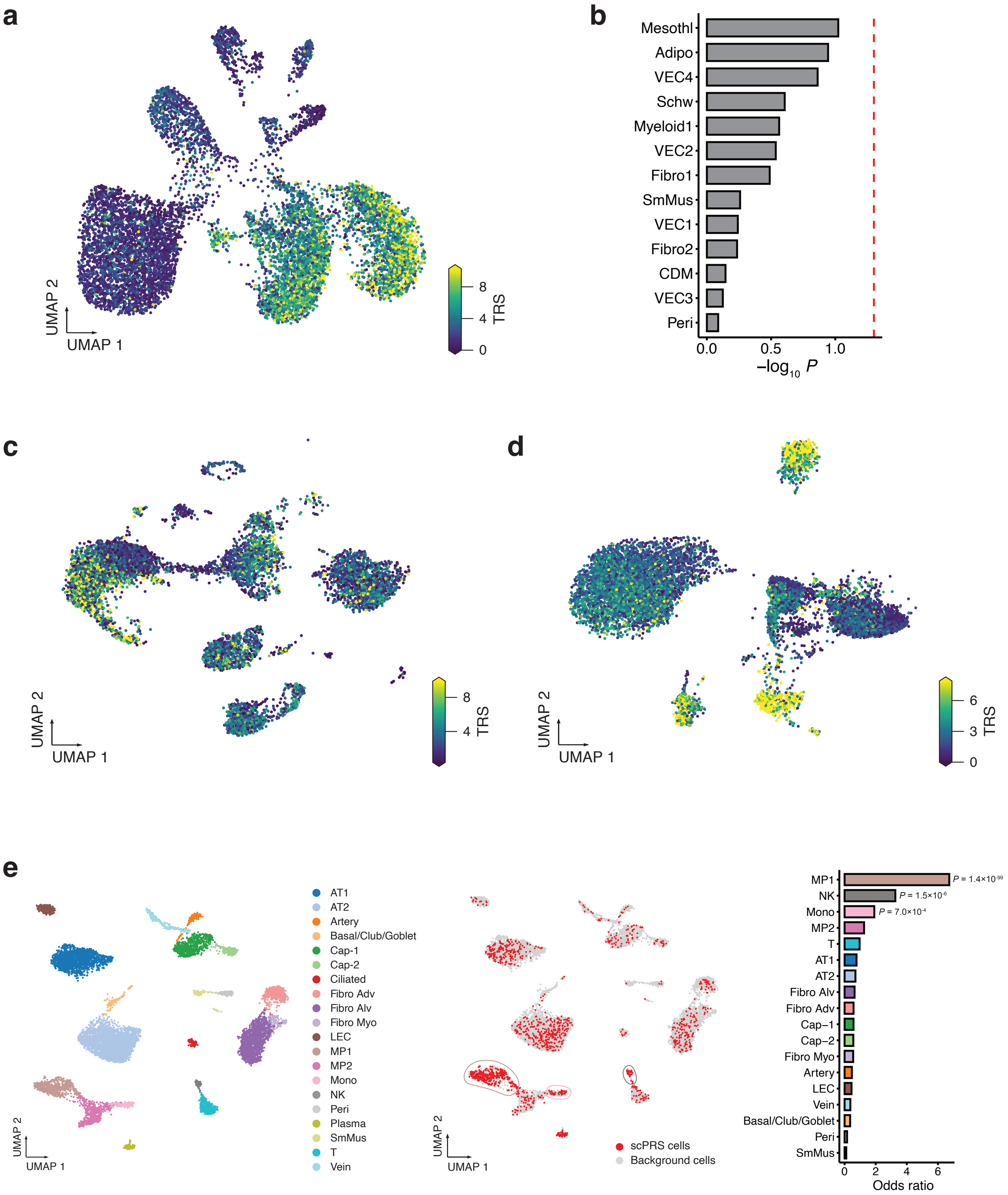
a
UMAP 2
UMAP 1
8
4
0
TRS
b
Mesothl
Adipo
VEC4
Schw
Myeloid1
VEC2
Fibro1
SmMus
VEC1
Fibro2
CDM
VEC3
Peri
0.0
0.5
1.0
$-\log_{10} P$
c
UMAP 2
UMAP 1
8
4
TRS
d
UMAP 2
UMAP 1
6
3
0
TRS
e
AT1
AT2
Artery
Basal/Club/Goblet
Cap-1
Cap-2
Ciliated
Fibro Adv
Fibro Alv
Fibro Myo
LEC
MP1
MP2
Mono
NK
Peri
Plasma
SmMus
T
Vein
UMAP 2
UMAP 1
scPRS cells
Background cells
MP1
NK
Mono
MP2
T
AT1
AT2
Fibro Alv
Fibro Adv
Cap−1
Cap−2
Fibro Myo
Artery
LEC
Vein
Basal/Club/Goblet
Peri
SmMus
$P = 1.4\times10^{-99}$
$P = 1.5\times10^{-6}$
$P = 7.0\times10^{-4}$
0
2
4
6
Odds ratio

**Supplementary Fig. 6 | Additional results on disease cell prioritization. a**, T2D-cell relevance computed by SCAVENGE. UMAP, uniform manifold approximation and projection; TRS, trait relevance score. **b**, The enrichment of HCM heritability across different cell types. The red dashed line indicates *P* = 0.05. *P*-value by stratified LDSC (sLDSC). Mesothl, mesothelial cell; Adipo, adipose cell; CDM, cardiomyocyte; Fibro, fibroblast; LEC, lymphatic endothelial cell; Peri, pericyte; Schw, Schwann cell; SmMus, smooth muscle cell; VEC, vascular endothelial cell. **c-d**, Disease-cell relevance computed by SCAVENGE for HCM (**c**) and AD (**d**), respectively. The same GWAS and single-cell datasets were used in SCAVENGE as those used in scPRS to ensure a fair comparison. **e,** Severe-COVID19-relevant cells prioritized by scPRS. The human lung single-cell dataset, along with annotated cell types, is shown in the left panel. UMAP, uniform manifold approximation and projection. Severe-COVID19-relevant cells prioritized by scPRS (in red) are shown in the middle panel. Cell clusters enriched with scPRS-prioritized cells are highlighted in closed curves with corresponding cell type colors. Enrichment of scPRS-selected cells within each cell type is shown in the right panel. *, adjusted *P* < 0.1. Odds ratio and *P*-value by one-sided Fisher's exact test. Cell type abbreviations: AT1, alveolar epithelial type I; AT2, alveolar epithelial type II; Cap, capillary endothelial cell; Fibro alv, alveolar fibroblast; Fibro myo, myofibroblast; Fibro adv, adventitial fibroblast; LEC, lymphatic endothelial cell; MP, macrophage; Mono, monocyte; NK, natural killer; Peri, pericyte; SmMus, smooth muscle.

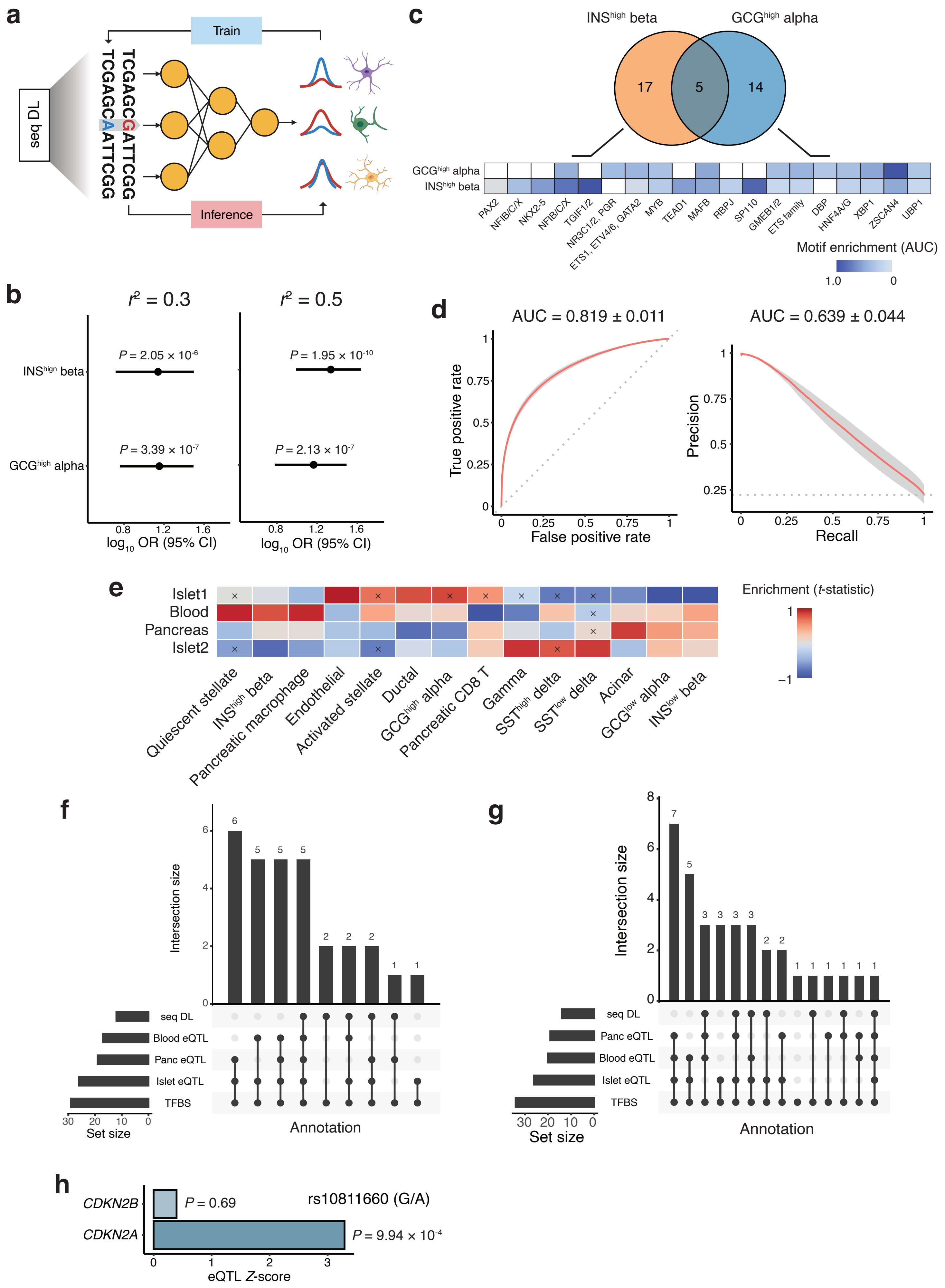
a
Train
seq DL
TCGAGCGATTCGG
TCGAGCAATTCGG
Inference
c
INS^high beta
GCG^high alpha
17
5
14
GCG^high alpha
INS^high beta
PAX2
NFIB/C/X
NKX2-5
NFIB/C/X
TGIF1/2
NR3C1/2, PGR
ETS1, ETV4/6, GATA2
MYB
TEAD1
MAFB
RBPJ
SP110
GMEB1/2
ETS family
DBP
HNF4A/G
XBP1
ZSCAN4
UBP1
Motif enrichment (AUC)
1.0
0
b
r² = 0.3
r² = 0.5
INS^hign beta
GCG^high alpha
P = 2.05 × 10^-6
P = 3.39 × 10^-7
P = 1.95 × 10^-10
P = 2.13 × 10^-7
0.8
1.2
1.6
log10 OR (95% CI)
d
AUC = 0.819 ± 0.011
AUC = 0.639 ± 0.044
True positive rate
False positive rate
Precision
Recall
0
0.25
0.5
0.75
1
e
Islet1
Blood
Pancreas
Islet2
Enrichment (t-statistic)
1
−1
Quiescent stellate
INS^high beta
Pancreatic macrophage
Endothelial
Activated stellate
Ductal
GCG^high alpha
Pancreatic CD8 T
Gamma
SST^high delta
SST^low delta
Acinar
GCG^low alpha
INS^low beta
f
Intersection size
6
5
5
5
2
2
2
1
1
seq DL
Blood eQTL
Panc eQTL
Islet eQTL
TFBS
30
20
10
0
Set size
Annotation
g
Intersection size
7
5
3
3
3
3
2
2
1
1
1
1
1
1
seq DL
Panc eQTL
Blood eQTL
Islet eQTL
TFBS
Set size
Annotation
h
CDKN2B
CDKN2A
P = 0.69
rs10811660 (G/A)
P = 9.94 × 10^-4
0
1
2
3
eQTL Z-score

**Supplementary Fig. 7 | Additional results of multiomic analysis for T2D. a**, Schematic of the sequence-based deep learning model. **b**, Enrichment of T2D-associated variants within cCREs that were differentially accessible in scPRS-prioritized cells. Two linkage disequilibrium (LD) thresholds, $r^2$ = 0.3 (left; $n$ = 1,352,052) and $r^2$ = 0.5 (right; $n$ = 1,881,838), were adopted in clumping. $P$-value by two-sided Fisher's exact test. The $\log_{10}$(OR) and 95% CI are annotated by the dot and error bar, respectively. OR, odds ratio; CI, confidence interval; cCRE, candidate cis-regulatory element. **c**, T2D-cCREs (top) and their motif enrichment (bottom) in two T2D-relevant cell types including $GCG^{high}$ alpha and $INS^{high}$ beta cells. Motif enrichment was measured by AUC. Row-wise standardization was performed. Only significant enrichment (adjusted $P$ < 0.1, Bonferroni correction) is colored. $P$-value by hypergeometric test. AUC, the area under the receiver operating characteristic (ROC) curve. **d**, The receiver operating characteristic curve (ROC; left) and the precision-recall curve (PRC; right) showing predictive performance of the sequence model across various cell types in the human pancreas. The red line and gray area represent the mean and 95% CI, respectively. Performance of a random predictor is shown in the dashed gray line. AUC, the area under the curve. **e**, Comparison of variant effect prediction scores between eQTLs and other variants across different tissues and cell types. Comparison was performed using two-sided $t$-test. ×, adjusted $P$ > 0.1 by Benjamini-Hochberg correction. Columns were standardized. **f-g**, Summary statistics of prioritized T2D risk variants in $GCG^{high}$ alpha cells (**f**) and $INS^{high}$ beta cells (**g**) using different annotations. TFBS, transcription factor binding site; Panc, pancreas. **h,** Islet eQTL result of rs10811660. The effect allele is G. $P$-value by eQTL analysis.

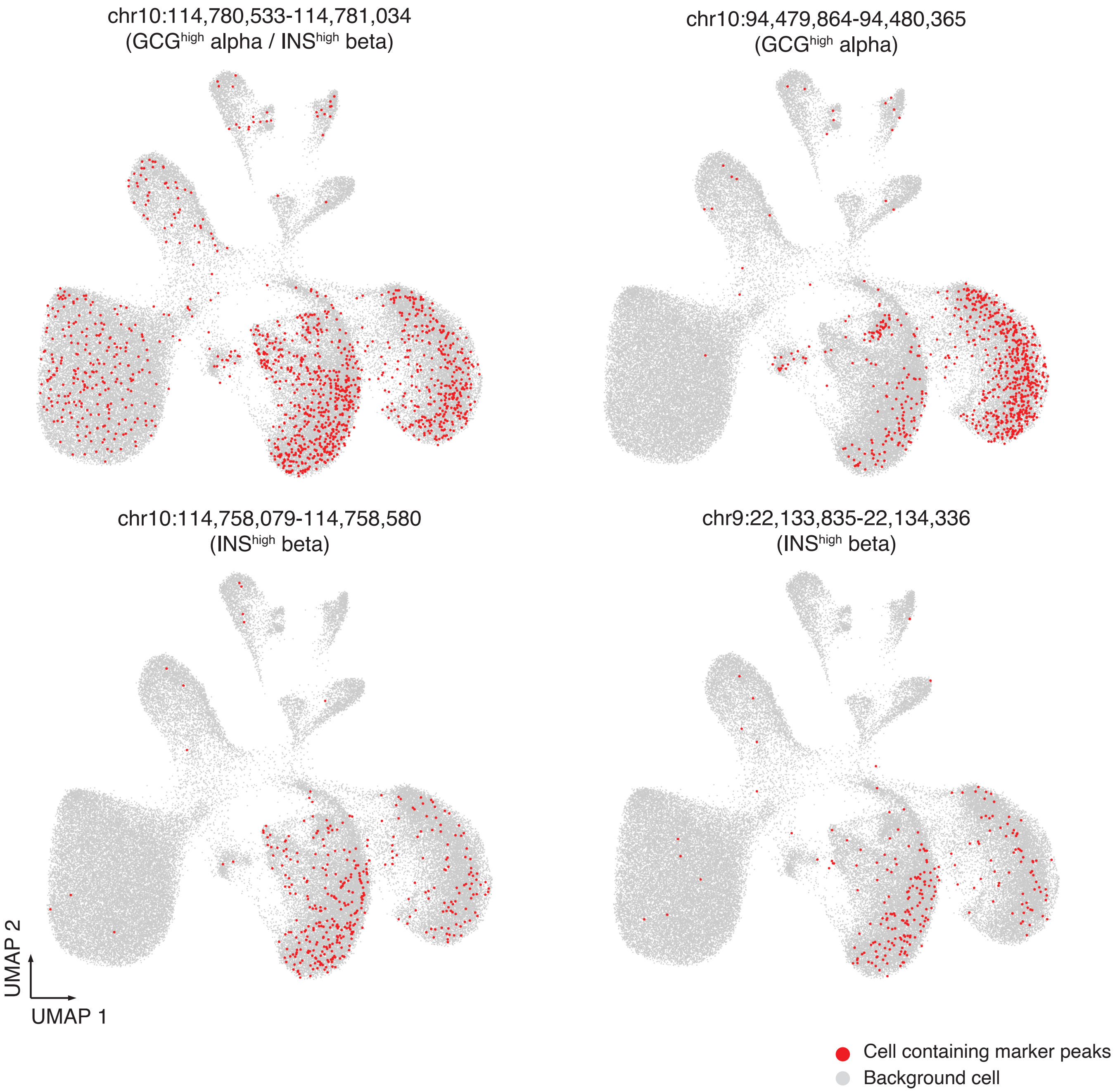


**Supplementary Fig. 8 | Cells containing marker peaks of scPRS-selected populations within GCG$^{high}$ alpha and INS$^{high}$ beta cells.** UMAP, uniform manifold approximation and projection.

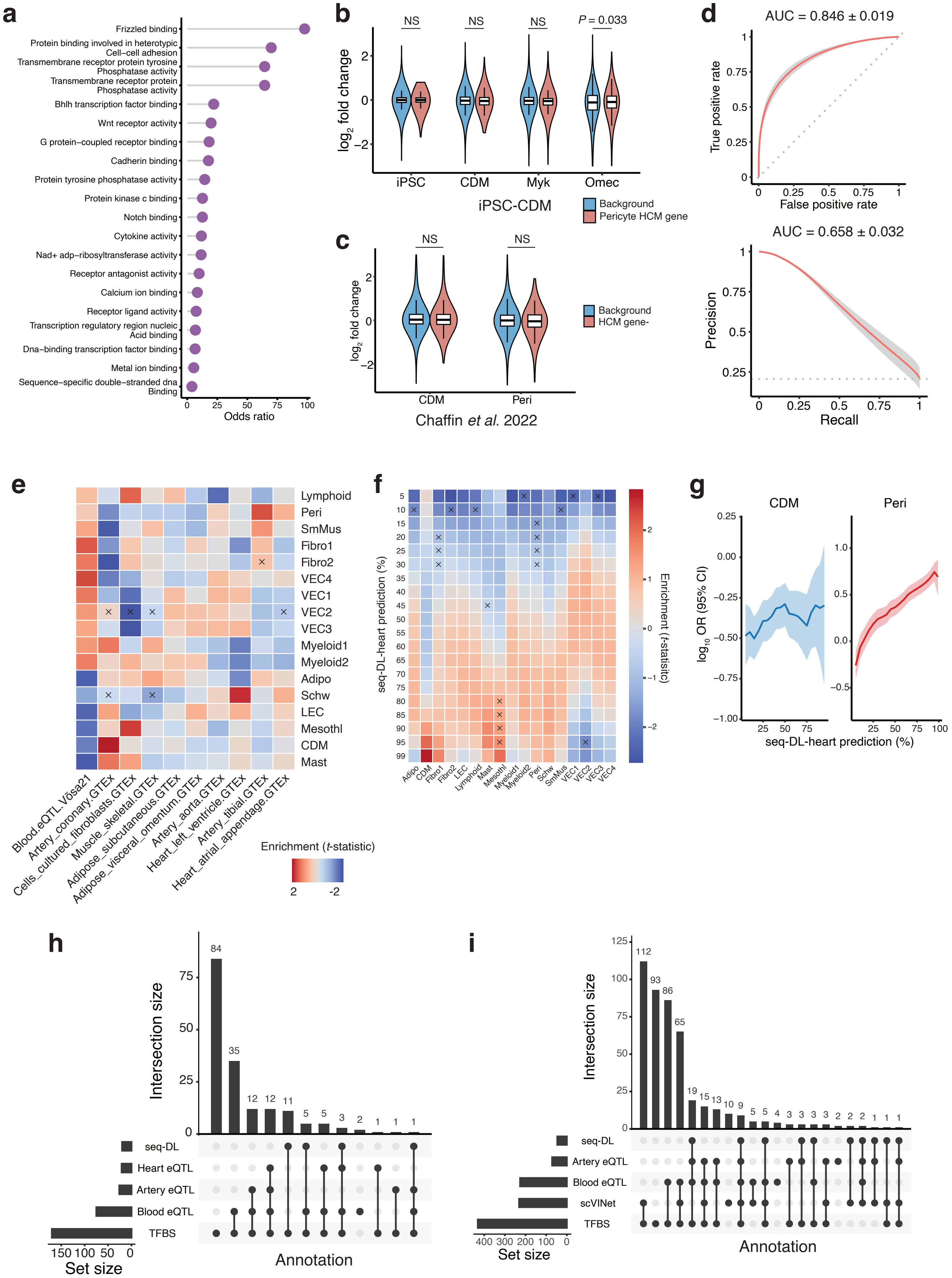
a
Frizzled binding
Protein binding involved in heterotypic
Cell–cell adhesion
Transmembrane receptor protein tyrosine
Phosphatase activity
Transmembrane receptor protein
Phosphatase activity
Bhlh transcription factor binding
Wnt receptor activity
G protein–coupled receptor binding
Cadherin binding
Protein tyrosine phosphatase activity
Protein kinase c binding
Notch binding
Cytokine activity
Nad+ adp–ribosyltransferase activity
Receptor antagonist activity
Calcium ion binding
Receptor ligand activity
Transcription regulatory region nucleic
Acid binding
Dna–binding transcription factor binding
Metal ion binding
Sequence–specific double–stranded dna
Binding
Odds ratio
b
NS
NS
NS
P = 0.033
log2 fold change
iPSC
CDM
Myk
Omec
iPSC-CDM
Background
Pericyte HCM gene
c
NS
NS
log2 fold change
CDM
Peri
Chaffin et al. 2022
Background
HCM gene-
d
AUC = 0.846 ± 0.019
True positive rate
False positive rate
AUC = 0.658 ± 0.032
Precision
Recall
e
Lymphoid
Peri
SmMus
Fibro1
Fibro2
VEC4
VEC1
VEC2
VEC3
Myeloid1
Myeloid2
Adipo
Schw
LEC
Mesothl
CDM
Mast
Blood.eQTL.Võsa21
Artery_coronary.GTEx
Cells_cultured_fibroblasts.GTEx
Muscle_skeletal.GTEx
Adipose_subcutaneous.GTEx
Adipose_visceral_omentum.GTEx
Artery_aorta.GTEx
Heart_left_ventricle.GTEx
Artery_tibial.GTEx
Heart_atrial_appendage.GTEx
Enrichment (t-statistic)
f
seq-DL-heart prediction (%)
Enrichment (t-statisitc)
Adipo
CDM
Fibro1
Fibro2
LEC
Lymphoid
Mast
Mesothl
Myeloid1
Myeloid2
Peri
Schw
SmMus
VEC1
VEC2
VEC3
VEC4
g
CDM
Peri
log10 OR (95% CI)
seq-DL-heart prediction (%)
h
Intersection size
seq-DL
Heart eQTL
Artery eQTL
Blood eQTL
TFBS
Set size
Annotation
i
Intersection size
seq-DL
Artery eQTL
Blood eQTL
scVINet
TFBS
Set size
Annotation

**Supplementary Fig. 9 | Additional results of multiomic analysis for HCM. a**, Lollipop chart of gene ontology (GO) enrichment (molecular function) for M16 genes. Significant GO terms (adjusted $P < 0.1$ by Benjamini-Hochberg (BH) correction) are shown. **b**, Expression fold change comparison between HCM pericyte genes and background transcriptome based on the HCM iPSC RNA-seq data. *P*-value by two-sided *t*-test ($n = 16{,}160$). The box plot center line, limits, and whiskers represent the median, quartiles, and 1.5x interquartile range (IQR), respectively. CDM, cardiomyocyte; Myk, Mavacamten; Omec, Omecamtiv mecarbil; iPSC, induced pluripotent stem cell; NS, not significant. **c**, Expression analysis of HCM cardiomyocyte and pericyte genes based on the pericyte and cardiomyocyte transcriptome data, respectively. *P*-value by two-sided *t*-test ($n = 11{,}683$). HCM gene- indicates CDM HCM gene in the context of pericyte data, and pericyte gene in the context of CDM data. The box plot center line, limits, and whiskers represent the median, quartiles, and 1.5x interquartile range (IQR), respectively. Peri, pericyte. **d**, The receiver operating characteristic curve (ROC; left) and the precision-recall curve (PRC; right) showing predictive performance of the sequence model across various cell types in the human left ventricle. The red line and gray area represent the mean and 95% CI, respectively. Performance of a random predictor is shown in the dashed gray line. AUC, the area under the curve. **e**, Comparison of variant effect prediction scores between eQTLs and other variants across different tissues and cell types. Comparison was performed using two-sided *t*-test. ×, adjusted $P > 0.1$ by BH correction. Rows were standardized. Mesothl, mesothelial cell; Adipo, adipose cell; CDM, cardiomyocyte; Fibro, fibroblast; LEC, lymphatic endothelial cell; Peri, pericyte; Schw, Schwann cell; SmMus, smooth muscle cell; VEC, vascular endothelial cell. **f**, Enrichment of transcription factor binding site (TFBS) disrupting variants within seq-DL-heart-prioritized variants (various thresholds applied). seq-DL-heart represents the sequence deep learning model trained on the left ventricle single-cell data. Enrichment was quantified by *t*-statistics. ×, adjusted $P > 0.1$ by BH correction. **g**, Enrichment of seq-DL-heart-prioritized HCM-associated variants within HCM-cCREs. OR, odds ratio. OR and CI by two-sided Fisher's exact test. The $\log_{10}$(OR) is annotated by the solid line and 95% CI is represented by the shaded area. **h-i**, Summary statistics of fine-mapped HCM risk variants in cardiomyocytes (**h**) and pericytes (**i**) using different annotations.

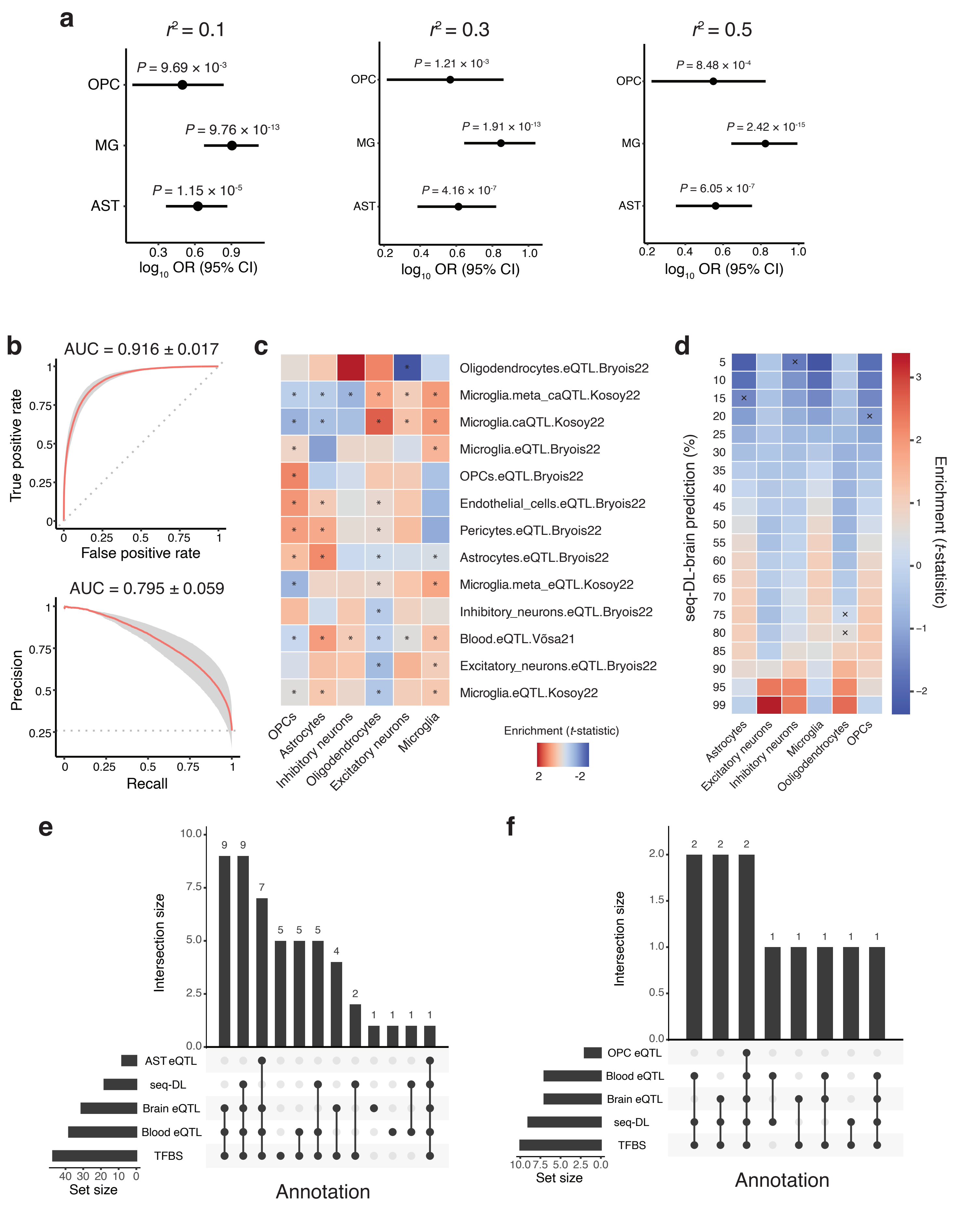

a
$r^2$ = 0.1
$r^2$ = 0.3
$r^2$ = 0.5
OPC
MG
AST
P = 9.69 × 10^-3
P = 9.76 × 10^-13
P = 1.15 × 10^-5
P = 1.21 × 10^-3
P = 1.91 × 10^-13
P = 4.16 × 10^-7
P = 8.48 × 10^-4
P = 2.42 × 10^-15
P = 6.05 × 10^-7
log10 OR (95% CI)
b
AUC = 0.916 ± 0.017
True positive rate
False positive rate
AUC = 0.795 ± 0.059
Precision
Recall
c
Oligodendrocytes.eQTL.Bryois22
Microglia.meta_caQTL.Kosoy22
Microglia.caQTL.Kosoy22
Microglia.eQTL.Bryois22
OPCs.eQTL.Bryois22
Endothelial_cells.eQTL.Bryois22
Pericytes.eQTL.Bryois22
Astrocytes.eQTL.Bryois22
Microglia.meta_eQTL.Kosoy22
Inhibitory_neurons.eQTL.Bryois22
Blood.eQTL.Võsa21
Excitatory_neurons.eQTL.Bryois22
Microglia.eQTL.Kosoy22
OPCs
Astrocytes
Inhibitory neurons
Oligodendrocytes
Excitatory neurons
Microglia
Enrichment (t-statistic)
d
seq-DL-brain prediction (%)
Enrichment (t-statisitc)
Astrocytes
Excitatory neurons
Inhibitory neurons
Microglia
Oolgodendrocytes
OPCs
e
Intersection size
AST eQTL
seq-DL
Brain eQTL
Blood eQTL
TFBS
Set size
Annotation
f
Intersection size
OPC eQTL
Blood eQTL
Brain eQTL
seq-DL
TFBS
Set size
Annotation

**Supplementary Fig. 10 | Additional results of multiomic analysis for AD. a**, Enrichment of AD-associated variants within cCREs that were differentially accessible in scPRS-prioritized cells. Different linkage disequilibrium (LD) thresholds, including $r^2$ = 0.1 (left; *n* = 627,420), $r^2$ = 0.3 (middle; *n* = 1,070,754), and $r^2$ = 0.5 (right; *n* = 1,515,425), were adopted in clumping. *P*-value by two-sided Fisher's exact test. The $\log_{10}$(OR) and 95% CI are annotated by the dot and error bar, respectively. AST, astrocyte; MG, microglia; OPC, oligodendrocyte progenitor cell; OR, odds ratio; CI, confidence interval; cCRE, candidate cis-regulatory element. **b**, The receiver operating characteristic curve (ROC; top) and the precision-recall curve (PRC; bottom) showing sequence deep learning model performance across various cell types in the human cortex. The red line and gray area represent the mean and 95% CI, respectively. The dashed gray line indicates the random prediction. AUC, the area under the curve. **c**, Comparison of variant effect prediction scores between eQTLs and other variants across different tissues and cell types. Comparison was performed using two-sided *t*-test. *, adjusted $P < 0.1$ by Benjamini-Hochberg (BH) correction. Rows were standardized. **d**, Enrichment of transcription factor binding site (TFBS) disrupting variants within seq-DL-brain-prioritized variants (various thresholds applied). seq-DL-brain, the sequence deep learning model trained on the cortex scATAC-seq data. Enrichment was quantified by *t*-statistics. ×, adjusted $P > 0.1$ by BH correction. **e-f**, Summary statistics of prioritized AD risk variants in astrocytes (**e**) and OPCs (**f**) using different annotations.

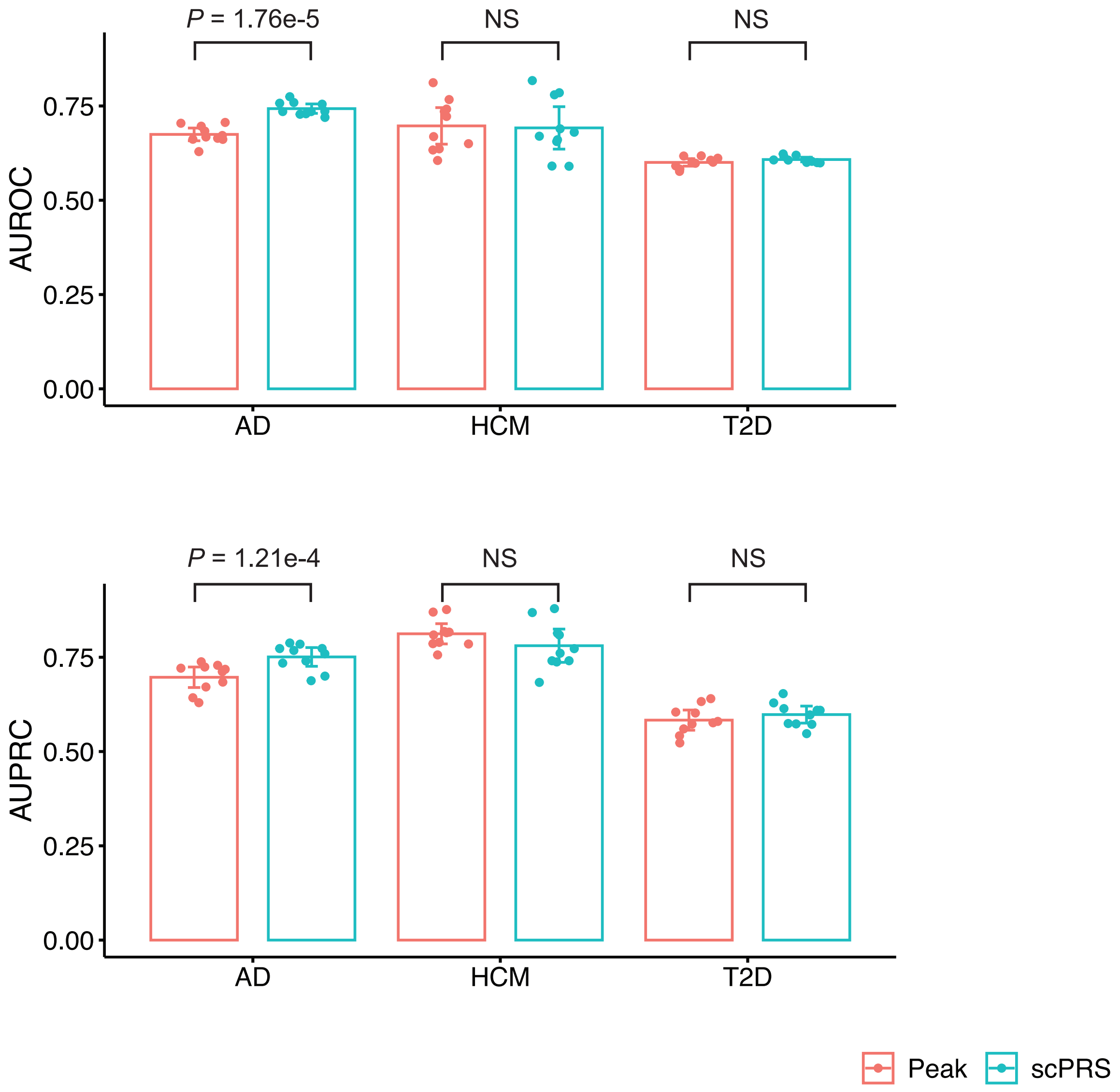


**Supplementary Fig. 11 | Predictive performance of C+T PRS based on variants selected by scPRS.** The training and testing procedure was conducted for 10 repeats with different random seeds. The bar plot and error bar represent the mean and the 95% confidence interval (CI), respectively. *P*-value by two-sided paired *t*-test. AUROC, area under the receiver operating characteristic curve; AUPRC, area under the precision-recall curve; NS, not significant; Peak, C+T PRS built on disease-associated variants (GWAS $P < 0.05$) within differentially accessible chromatin regions in scPRS-prioritized cells.
