# Supplementary material for: Single-cell polygenic risk scores dissect cellular and molecular heterogeneity of complex human diseases
Source: Nat Biotechnol. 2025 Jul 25;44(5):845–61. doi: 10.1038/s41587-025-02725-6 (PMC13180658; doi:10.1038/s41587-025-02725-6)
Supplement: Supplementary file 2 — Reporting Summary [file 41587_2025_2725_MOESM2_ESM.pdf]

nature portfolio

Corresponding author(s): Michael Snyder

Last updated by author(s): May 21, 2025

Reporting Summary

Nature Portfolio wishes to improve the reproducibility of the work that we publish. This form provides structure for consistency and transparency in reporting. For further information on Nature Portfolio policies, see our Editorial Policies and the Editorial Policy Checklist.

Statistics

For all statistical analyses, confirm that the following items are present in the figure legend, table legend, main text, or Methods section.

n/a	Confirmed	
☐	☒	The exact sample size (*n*) for each experimental group/condition, given as a discrete number and unit of measurement
☐	☒	A statement on whether measurements were taken from distinct samples or whether the same sample was measured repeatedly
☐	☒	The statistical test(s) used AND whether they are one- or two-sided<br>*Only common tests should be described solely by name; describe more complex techniques in the Methods section.*
☐	☒	A description of all covariates tested
☐	☒	A description of any assumptions or corrections, such as tests of normality and adjustment for multiple comparisons
☐	☒	A full description of the statistical parameters including central tendency (e.g. means) or other basic estimates (e.g. regression coefficient) AND variation (e.g. standard deviation) or associated estimates of uncertainty (e.g. confidence intervals)
☐	☒	For null hypothesis testing, the test statistic (e.g. *F*, *t*, *r*) with confidence intervals, effect sizes, degrees of freedom and *P* value noted<br>*Give P values as exact values whenever suitable.*
☒	☐	For Bayesian analysis, information on the choice of priors and Markov chain Monte Carlo settings
☒	☐	For hierarchical and complex designs, identification of the appropriate level for tests and full reporting of outcomes
☐	☒	Estimates of effect sizes (e.g. Cohen's *d*, Pearson's *r*), indicating how they were calculated

Our web collection on statistics for biologists contains articles on many of the points above.

Software and code

Policy information about availability of computer code

Data collection	dx-toolkit v0.347.0 was used to download data from UKBB RAP.
Data analysis	The sequence deep learning model was implemented using Selene v0.2.0 (https://github.com/FunctionLab/selene). Baseline PRS methods included C+T (PLINK v1.9), LDpred2 (bigsnpr v1.12.2), and Lassosum v0.4.5. We also implemented SCAVENGE v1.0.2 (https://github.com/sankaranlab/SCAVENGE) and stratified LDSC v1.0.1 (https://github.com/bulik/ldsc). Single-cell data analysis was performed using Scrublet v0.2.2, Seurat v4.3.0, Scanpy v1, Signac v1.11.0, and ALLCools v1.1.0 (https://github.com/lhqing/ALLCools). GO analysis was conducted using Enrichr (https://maayanlab.cloud/Enrichr/) if not specified. TF motif analysis was carried out using GimmeMotifs v0.18.0(https://gimmemotifs.readthedocs.io/en/master/). We performed TF binding site prediction using SNP2TFBS (https://epd.expasy.org/snp2tfbs/) and motifbreakR v2.15.5 (https://github.com/Simon-Coetzee/motifBreakR). All statistical analyses were performed using Python v3 and R v4. scPRS source code can be found at https://github.com/szhang1112/scPRS.

For manuscripts utilizing custom algorithms or software that are central to the research but not yet described in published literature, software must be made available to editors and reviewers. We strongly encourage code deposition in a community repository (e.g. GitHub). See the Nature Portfolio guidelines for submitting code & software for further information.

Data

Policy information about availability of data

All manuscripts must include a data availability statement. This statement should provide the following information, where applicable:

- Accession codes, unique identifiers, or web links for publicly available datasets
- A description of any restrictions on data availability
- For clinical datasets or third party data, please ensure that the statement adheres to our policy

The PBMC multiome dataset is available from 10x Genomics (https://support.10xgenomics.com/single-cell-multiome-atac-gex/datasets/1.0.0/pbmc_granulocyte_sorted_10k). The single-cell multiome data (snRNA-seq and snATAC-seq coassay) of the human left ventricle and lung are publicly accessible through ENCODE 4 (https://www.encodeproject.org/single-cell/?type=Experiment&assay_slims=Single+cell&status=released). All other single-cell ATAC-seq datasets were obtained from their original publications (refs. 7,8). The WGS data used in simulation are available from ref. 28. Individual-level genotype-phenotype data for T2D and AD were sourced from the UK Biobank. The WGS and iPSC RNA-seq data for HCM are available from ref. 14. The COVID-19 WGS and clinical data are available upon request from the corresponding authors (P.S.T. and M.P.S.); these data are not publicly available due to US Government and Department of Veteran's Affairs restrictions relating to participant privacy and consent. The independent target cohorts for T2D, HCM, and AD are accessible through EGA (no. EGAD00001002247), UKBB, and ADNI (https://adni.loni.usc.edu/data-samples/adni-data/), respectively. The HCM snRNA-seq dataset was obtained from ref. 52. All GWAS summary statistics data were acquired from their original publications (refs. 27,53–55). The GTEx and islet eQTL datasets were downloaded from the eQTL Catalogue (https://www.ebi.ac.uk/eqtl/). Other eQTL and caQTL datasets were obtained from their original publications (refs. 56–59). The reference human genomes (hg19 and hg38) are available from https://hgdownload.soe.ucsc.edu/downloads.html#human.

Research involving human participants, their data, or biological material

Policy information about studies with human participants or human data. See also policy information about sex, gender (identity/presentation), and sexual orientation and race, ethnicity and racism.

Reporting on sex and gender	Sex was included as a covariate in our analysis when possible.
Reporting on race, ethnicity, or other socially relevant groupings	We focused on European samples when possible. Mixed populations were analyzed for small cohorts such as HCM and COVID-19. The first 10 genotype principal components (PCs) were included as covariates in the analysis.
Population characteristics	The UK Biobank is a large-scale prospective cohort study including 500,000 participants recruited between 2006 and 2010. These participants, aged between 40 and 69, provided extensive biological and medical data, including physical measures, lifestyle information, and health records. We constructed T2D and AD target cohorts based on the UKBB with matched age and sex between cases and controls. Cases were defined by the ICD-10 code in inpatient record, death record, or diagnosis summary record.<br><br>HCM target cohort includes 97 healthy controls (age: 52.4 [mean] ± 18.2 [SD]; male: 54.6%) and HCM cases (age: 54.4 ± 16.3; male: 61.4%). The targeted patient population were patients with various cardiac procedures and non-cardiac patients with genetic conditions in clinic who were identified to us by their clinical providers. Non-cardiac patients were recruited in person during onsite clinic days or over the phone with permission by the providers. Healthy volunteers were recruited from our cardiovascular prevention clinic (i.e., patients with no diagnosis of heart disease).<br><br>The VA COVID-19 cohort was derived from the VA Million Veteran Program (MVP). COVID-19 cases were identified using an algorithm developed by the VA COVID National Surveillance Tool based on reverse transcription polymerase chain reaction laboratory test results conducted at VA clinics, supplemented with natural language processing on clinical documents for SARS-CoV-2 tests conducted outside of the VA19. We defined severe COVID-19 cases as patients who were hospitalized, received acute care, stayed in ICU, or deceased, and controls as those who did not meet these criteria. To minimize potential confounders, we restricted our analysis to non-elderly individuals (age < 65).
Recruitment	The recruitment of the HCM cohort is part of our CIRM cardiomyopathy project (ref. 14). The targeted patient population were patients with various cardiac procedures and non-cardiac patients with genetic conditions in clinic who were identified to us by their clinical providers. Non-cardiac patients were recruited in person during onsite clinic days or over the phone with permission by the providers. Healthy volunteers were recruited from our cardiovascular prevention clinic (i.e., patients with no diagnosis of heart disease).<br><br>The VA COVID-19 cohort was derived from the VA Million Veteran Program (MVP). The VA MVP is an ongoing national voluntary research program that aims to better understand how genetic, lifestyle, and environmental factors influence veteran health. Briefly, individuals aged 18 to over 100 years old have been recruited from over 60 VA Medical Centers nationwide since 2011 with current enrollment at >800,000. Informed consent is obtained from all participants to provide blood for genomic analysis and access to their full electronic health record (EHR) data within the VA prior to and after enrollment. The study received ethical and study protocol approval from the VA Central Institutional Review Board in accordance with the principles outlined in the Declaration of Helsinki. COVID-19 cases were identified using an algorithm developed by the VA COVID National Surveillance Tool based on reverse transcription polymerase chain reaction laboratory test results conducted at VA clinics, supplemented with natural language processing on clinical documents for SARS-CoV-2 tests conducted outside of the VA.
Ethics oversight	Ethics approval for the UKB study was obtained from the North West Centre for Research Ethics Committee (protocol no. 11/NW/0382). The Stanford CIRM cardiomyopathy project is in compliance with the Stanford Human Research Protection Program guidelines and approved by the Stanford Institutional Review Board (IRB #30064). In addition, the procedures are in

compliance with the International Society of Stem Cell Research guidelines and approved by the Stanford IRB/Stem Cell Research Oversight panel (SCRO #656). Ethics approval for the MVP COVID-19 study was obtained from the Office of Research and Development, Veterans Health Administration (MVP001)

Note that full information on the approval of the study protocol must also be provided in the manuscript.

Field-specific reporting

Please select the one below that is the best fit for your research. If you are not sure, read the appropriate sections before making your selection.

☒ Life sciences ☐ Behavioural & social sciences ☐ Ecological, evolutionary & environmental sciences

For a reference copy of the document with all sections, see nature.com/documents/nr-reporting-summary-flat.pdf

Life sciences study design

All studies must disclose on these points even when the disclosure is negative.

Sample size	All disease cases were utilized in our analysis for each cohort. The same number of control samples were randomly selected from UKBB for T2D and AD.
Data exclusions	Samples that failed quality controls were excluded from the analysis. For severe COVID-19, samples with age >= 65 were excluded to remove confounding factors. Overweight individuals (body mass index (BMI) >= 25) were excluded for T2D.
Replication	The siRNA experiment was replicated for 8 times for each siRNA. The prime-editing experiment was replicated for 4 times. All replicates were successful.
Randomization	Random training and testing dataset splits were employed in evaluating model performance.
Blinding	In PRS model training, investigators were not blinded to group allocation because the sample labels were needed for training an ML model. In other scenarios, such as model evaluation and siRNA/prime-editing experiments, the investigators were blinded to group allocation.

Reporting for specific materials, systems and methods

We require information from authors about some types of materials, experimental systems and methods used in many studies. Here, indicate whether each material, system or method listed is relevant to your study. If you are not sure if a list item applies to your research, read the appropriate section before selecting a response.

Materials & experimental systems

n/a	Involved in the study	
☒	☐	Antibodies
☐	☒	Eukaryotic cell lines
☒	☐	Palaeontology and archaeology
☒	☐	Animals and other organisms
☒	☐	Clinical data
☒	☐	Dual use research of concern
☒	☐	Plants

Methods

n/a	Involved in the study	
☒	☐	ChIP-seq
☒	☐	Flow cytometry
☒	☐	MRI-based neuroimaging

Eukaryotic cell lines

Policy information about cell lines and Sex and Gender in Research

Cell line source(s)	For prime editing experiment, microglia were derived from the WTC11 iPSC line, which was originally established in Dr. Bruce Conklin's lab at UCSF and later shared as a gift with Dr. Yin Shen's lab. Detailed information about this iPSC line can be found in Miyaoka et al., Nature Methods (PMID: 24509632) and at http://hpscreg.eu/cell-line/UCSF1001-A.<br><br>For TMEM119-tdTomato reporter iPSCs, lymphoblastoid cells obtained from a healthy, 56-year-old male, were provided by the NINDS biorepository and reprogrammed into iPSCs in house.
Authentication	hPSCs are routinely tested for expressing stem cell markers and differentiated microglia are tested by know microglia markers. Confirmation of the knock-in TMEM119-tdTomato construct was obtained by genotyping.
Mycoplasma contamination	All cells used in the present study were verified as mycoplasma contamination free.
Commonly misidentified lines<br>(See ICLAC register)	None of the cell lines used are commonly misidentified lines.

Plants

Seed stocks	NA
Novel plant genotypes	NA
Authentication	NA
